# Rapid Approximate Subset-Based Spectra Prediction for Electron Ionization–Mass Spectrometry

**DOI:** 10.1021/acs.analchem.2c02093

**Published:** 2023-01-25

**Authors:** Richard Licheng Zhu, Eric Jonas

**Affiliations:** † Committee on Computational and Applied Mathematics, Department of Statistics, University of Chicago, 5747 South Ellis Avenue, Chicago, Illinois60637, United States; ‡ Department of Computer Science, University of Chicago, 5730 South Ellis Avenue, Chicago, Illinois60637, United States

## Abstract

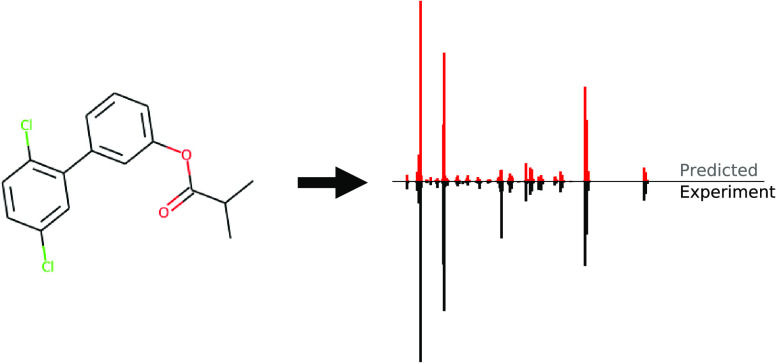

Mass spectrometry is a vital tool in the analytical chemist’s toolkit, commonly used to identify the presence of known compounds and elucidate unknown chemical structures. All of these applications rely on having previously measured spectra for known substances. Computational methods for predicting mass spectra from chemical structures can be used to augment existing spectral databases with predicted spectra from previously unmeasured molecules. In this paper, we present a method for prediction of electron ionization–mass spectra (EI–MS) of small molecules that combines physically plausible substructure enumeration and deep learning, which we term rapid approximate subset-based spectra prediction (RASSP). The first of our two models, *FormulaNet*, produces a probability distribution over chemical subformulae to achieve a state-of-the-art forward prediction accuracy of 92.9% weighted (Stein) dot product and database lookup recall (within top 10 ranked spectra) of 98.0% when evaluated against the NIST 2017 Mass Spectral Library. The second model, *SubsetNet*, produces a probability distribution over vertex subsets of the original molecule graph to achieve similar forward prediction accuracy and superior generalization in the high-resolution, low-data regime. Spectra predicted by our best model improve upon the previous state-of-the-art spectral database lookup error rate by a factor of 2.9×, reducing the lookup error (top 10) from 5.7 to 2.0%. Both models can train on and predict spectral data at arbitrary resolution. Source code and predicted EI–MS spectra for 73.2M small molecules from PubChem will be made freely accessible online.

## Introduction

Mass spectrometry (MS) provides valuable information about chemical substances, enabling scientists to understand chemical abundance, identity, and certain structural motifs. Gas chromatography/electron ionization–mass spectrometry (GC/EI–MS) is a highly reproducible and cost-effective version of MS that is used across fields such as medicine,^1,2^ ecology,^3^ protein sequencing,^4^ metabolomics,^5^ and more. For these reasons, GC/EI–MS spectral databases have grown significantly over the past few decades. Experimentally obtained spectra can be compared to databases of known spectra to identify and understand the structure of molecules, but the limited coverage of these databases hinders their use. By augmenting these spectral libraries using in silico methods for spectral prediction, scientists may be able to perform real-time identification of unknown substances by comparing experimentally obtained spectra of novel substances to massive chemical libraries consisting of both measured and predicted spectra.

**Figure 1:**
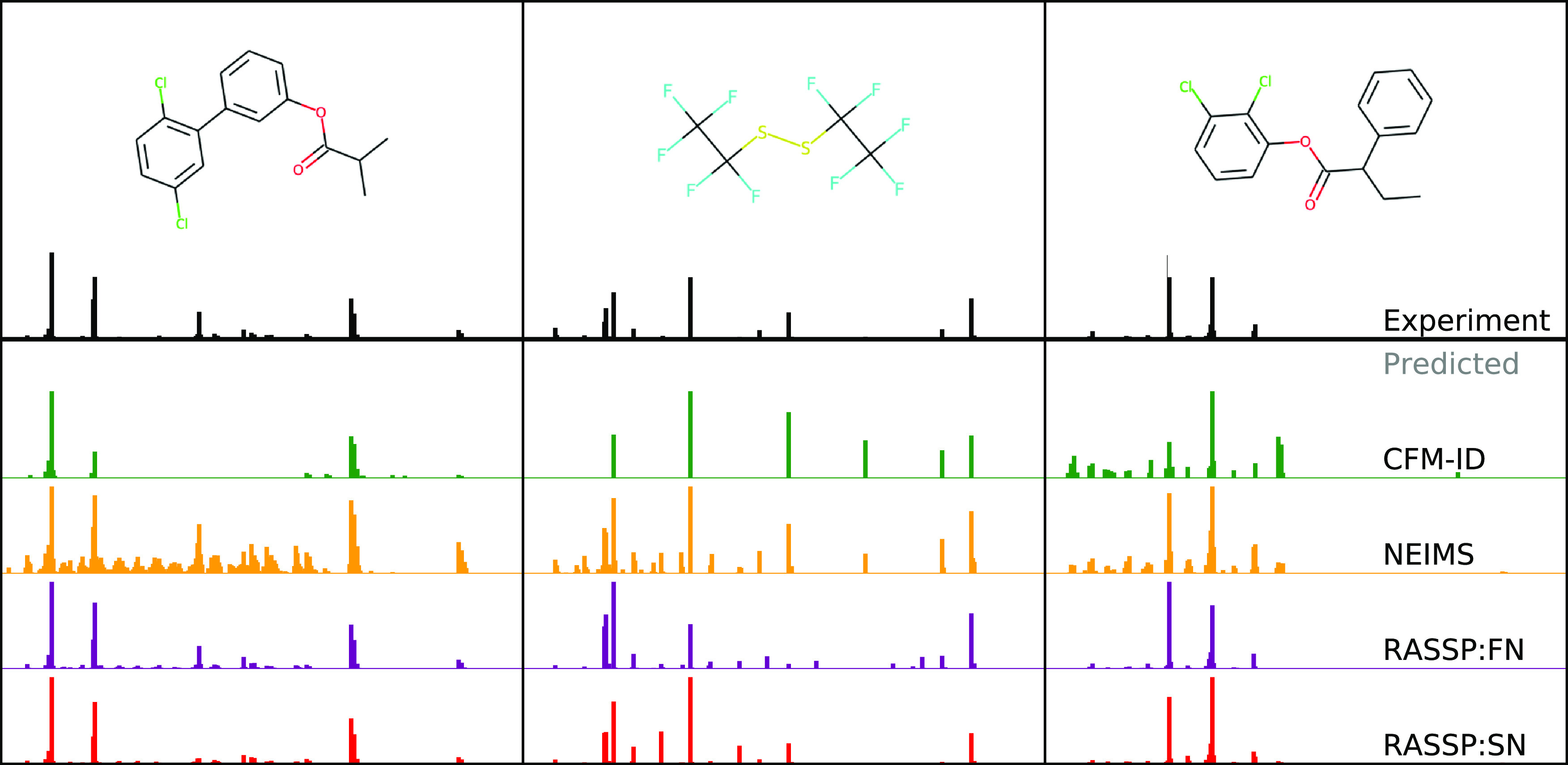
Example predictions on the held out NIST 2017 test set from the models we assess in this paper: FormulaNet (RASSP:FN), SubsetNet (RASSP:SN), NEIMS,^7^ CFM-ID,^8^ and experimentally measured spectra.^9^

In this paper, we present two state-of-the-art models for in silico prediction of EI–MS spectra on small molecules. Our approach, which we call “rapid approximate subset-based spectra prediction” (RASSP) predicts probability distributions over reduced representations of molecular fragments—atom subsets (vertex subsets of the molecular graph) and chemical formulae. By leveraging existing spectral databases, enumerating physically plausible substructures, and using deep learning to estimate probability distributions over these substructures, we outperform previous methods for spectral prediction by a significant margin. We evaluate these models on spectral similarity metrics^6^ and a practical database lookup task.^7^ Our first model, *FormulaNet* (hereafter RASSP:FN) predicts probability distributions over possible chemical subformulae and achieves a state-of-the-art forward prediction accuracy of 92.9% Stein dot product^6^ and a database lookup recall (at 10) of 98.0%. Our second model, *SubsetNet* (hereafter RASSP:SN) predicts probability distributions over atom subsets and achieves a forward prediction accuracy of 91.8% Stein dot product and a database lookup recall (at 10) of 95.2%. Notably, *RASSP:SN* outperforms *RASSP:FN* in the high-resolution, low-data regime, indicating that it may be useful for future high-resolution (sub-Dalton resolution) CID-based MS2 data. Direct comparison against two previous methods for in silico spectral prediction demonstrates that our best model improves the lookup error rate over the prior best forward model by a factor of ∼3×, reducing lookup error rate (at 10) from over 6 to 2%, approaching the limiting error rate associated with experiment-to-experiment noise of 1.2% (Figure 1).

## Background

Gas chromatography/electron ionization–mass spectrometry (GC/EI–MS) ionizes a volatile substance via high-energy electron bombardment. The subsequent relaxation of the ionized substance from the high-energy state induces fragmentation, generating a shower of charged and neutral fragments. The charge-to-mass (*m*/*z*) ratio of the fragments is then measured in a spectrometer. It is reasonable to assume that the fragments are singly charged,^7,8^ so the measured *m*/*z* values can be interpreted directly as fragment masses. Due to the cost-effectiveness and experimental reproducibility of GC/EI–MS, it is a mainstay of modern analytical chemistry workflows. The spectrum of a given compound is commonly used as a “fingerprint” used for matching against known database spectra. Additionally, it is often used as one of the first steps in structural characterization.

Currently, the NIST Mass Spectral Library^9^ is the largest publicly available database of EI–MS spectra, containing over 300,000 spectra for molecules containing ≤128 atoms. However, the space of possible molecules is incredibly large, and even annotated databases such as PubChem^10^ have over 100 million known chemical structures. Less than 0.30% of the PubChem compounds have measured spectra. Clearly, experimental characterization at such a scale is prohibitive. This is exacerbated by the fact that cheap products are easily attainable and measured many times, while many of the structures in PubChem come from the long tail of rare, non-natural, or difficult-to-procure set of compounds. Such limitations require computational and statistical approaches to predicting mass spectra.

Computational approaches to the mass spectral prediction problem fall into two categories: first-principles physical-based simulation and data-driven statistical methods.

### First-Principles Physical Simulation

#### Purely Statistical Theories

Ab initio approaches to EI–MS prediction leverage quasi-equilibrium theory (QET) or Rice–Ramsperger–Kassel–Marcus (RRKM) theories,^11^ which explicitly model the redistribution of the energy over the internal degrees of freedom. By keeping only the relevant vibrational modes (with a harmonic oscillator approximation), the density of states (core to the estimation of the rate constants) may be approximated. Such theories and their expansions have been used to study the relative abundances of fragment ions in well-known spectra.^12^ The need to enumerate the possible reaction pathways limits the successful application of such theories to very small molecules.

#### Born–Oppenheimer Molecular Dynamics

Methods such as QCEIMS and its derivatives^13,14^ combine quantum-mechanical Born–Oppenheimer molecular dynamics (MD) with fragmentation pathways to compute fragment ions within picosecond reaction times and femtosecond intervals for the MD trajectories. Statistical sampling of these trajectories then provides a distribution of observed fragments, generating a spectrum. However, even with the approximations made to reduce runtime, the runtime complexity is prohibitive for scaling, on the order of *O* (100 h) for small molecules less than 100 Da in mass.^14^ While these methods can often qualitatively identify plausible fragmentation pathways, their accuracy is not yet high enough for compound identification.^13^

### Data-Driven Statistical Methods

Computational systems for predicting mass spectra fragmentation were a topic of interest for early AI researchers, leading to projects like DENDRAL^15^ in the 1960s, which applied rule-based heuristic programming to the structural elucidation in organic chemistry. The heuristics used in the project have been improved upon over the last few decades, as chemists continue to add to a library of known fragmentation processes,^11^ by which chemical bonds and atoms are broken and rearranged. These heuristics are used by chemists to manually identify and explain the occurrence of particular peaks in small-molecule EI–MS spectra.^16^

Early approaches were rule-based approaches, iteratively applying thousands of known rules to combinatorially enumerate possible fragments. Such methods have very high recall, providing a possible explanation for every peak in a spectrum. Recent work fuses the high recall of the combinatorial approach with learned models to improve precision. In particular, the series of CFM-ID papers^8,17−19^ achieved state-of-the-art results in using a general rule-based fragmentation scheme to generate a large fragmentation tree for each molecule and then investigated the parameters for a model that parameterizes a Markov transition process over the tree.

The advent of machine learning and graph neural networks has renewed the interest in this problem. Recent work^7,20^ innovates in this area using deep neural networks that directly predict spectra from molecular fingerprints or molecular graphs. These systems have been shown to do quite well on learning the regularities present in EI–MS data, achieving performance surpassing that of simpler linear or neural network models.

## Methods

The complete calculation of the full fragmentation tree for a given molecule undergoing EI–MS would contain all necessary information to accurately predict the observed spectrum: simply compute the isotopic *m*/*z* distribution for each observed fragment and sum these over all fragments weighting by the fragment probability. However, the physical complexities and possible fragmentation paths make this a very challenging, and perhaps impossible, computational task. Approaches like CFM-ID^17^ attempt to model this process, but the exponential growth in possible fragmentations naturally limits the types of fragmentation events and fragmentation tree depth, impacting spectral prediction accuracy.

**Figure 2:**
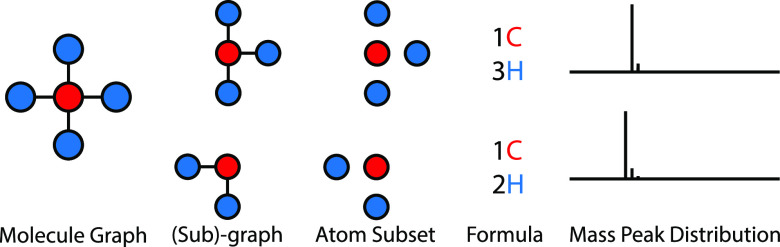
Different representation levels for the mass spectrometric forward problem. Each molecule is represented as a graph where nodes are atoms and edges are bonds. Subgraphs are connected components of the original graph, where both atom/bond presence in the subgraph is considered. Atom subsets are another level of abstraction, where only atom presence in the set is considered. Formulae are yet another level, where only the counts of unique elements are considered. Finally, each unique formula corresponds to a known mass peak distribution.

We instead reason backward from our observation: the spectrum. While one could attempt to directly predict the spectrum, given an input molecular structure or molecular fingerprint (like NEIMS), this discards effectively all physical intuition about the problem. As we state later, we are interested in developing methods that will naturally extend to higher-resolution spectra, and contemporary machine learning methods can struggle with extremely high-dimensional output spaces. Figure 2 illustrates the possible representation levels at which one can reason about the problem, starting from the input molecule structure (viewed as a graph) and ending with the mass peak distribution as viewed in the spectrometer.

Note that for any fragment child ion of the original molecule, both the chemical subformulae and the vertex (atom) subsets allow us to exactly determine the observed peak *m*/*z* distribution of the fragment. However, there are far fewer formulae than atom subsets and far fewer atom subsets than possible subgraphs. For example, C_6_H_12_O_6_ has a total of 18 bonds. If we consider complete bond breakages out to depth *d*, we can generate 18!/(18 – *d*)! unique bond breaks and up to the same amount of possible subgraphs but only 7 × 13 × 7 = 637 possible subformulae. For *d* ≥ 3, the number of possible subgraphs is already larger than the number of possible subformulae. Thus, we focus only on chemical formulae and atom subsets. Motivated by the need to generalize to higher-resolution spectra, we adopt two different physically informed substructure enumeration methods: one that produces possible fragment formulae (used in RASSP:FN) and another that produces possible fragment vertex subsets (used in RASSP:SN).

### Generating Subformulae

Generating subformulae for a given molecule is straightforward. For a given molecule, we can iteratively generate all subformulae by recursively taking the setwise Cartesian product of the possible subformulae for a single element of the molecule with the subformulae over the rest of the molecule. For example, getSubformulae (C_6_H_12_O_6_) = getSubformulae (C_6_) ⊗ getSubformulae (H_12_O_6_). The base case is a single element *X* occurring *N* times, where the possible subformulae are simply the possible occurrences of *X*: getSubformulae (*X*_*N*_) = [*X*_0_, *X*_1_, ···, *X*_*N*_]. However, only considering the chemical formula (which elements are present and how many) discards vital structural information such as bond connectivity. In doing so, we ignore all information about which formulae might appear more often in the final spectrum than others.

We thus explore an additional, richer representation of fragments: vertex (atom) subsets. We use atom subsets and vertex subsets interchangeably to refer a subset of the atoms present in a molecule. Atom subsets are preferred to complete fragment subgraphs because considering bond connectivity explodes the number of subgraph objects we must consider. Note that two fragment subgraphs with different bonds may still implicate the same subset of atoms from the original molecule.

Unfortunately, for most interesting molecules, it is quite infeasible to enumerate all possible subsets as a molecule with *N* atoms can have 2^*N*^ possible atomic subsets. Conveniently for us, this space of atomic subsets is highly redundant, with many atomic subsets having similar mass peaks in a spectrum. Thus, we cannot proceed like we did with the chemical subformulae earlier, where we could simply enumerate all possible subformulae. For atom subsets, we need to devise a scheme that can generate sufficiently plausible subsets. It should have enough generality to output all peaks in a spectrum but not so many as to be computationally intractable to fit a model later on.

### Generating Subsets

To select plausible subsets from this much larger space of possible subsets, we adopt a heuristic bond-breaking approach where we begin with an initial molecule and recursively break all possible bonds out to a particular depth. In this paper, we consider all fragments generated by breaking bonds out to *d* = 3. Discussion on why *d* = 3 is selected is presented later in the Evaluating the Impact of the Subset Enumeration section. To improve the recall of this process, we also perform exhaustive hydrogen rearrangements, a well-studied transition in mass spectrometric fragmentations.^11^ Since our fragment generation process features bond removal and addition, it is possible to generate subgraphs that are not subisomorphic to the original molecule graph. However, our process notably misses important fragmentation processes. Consider the fragmentation process for toluene. Toluene (C_7_H_8_) starts with a six-carbon-ring ion and one of the possible pathways leads to an intermediate seven-carbon ring ion. Such a graph structure is not isomorphic to the original graph and must be formed by the bonds breaking and rearranging to form a new ring ion.^11^ This fragment is not explicitly generated by our subset enumeration process, though the chemical formulae may still be output by our exhaustive formula enumeration.

### Graph Neural Networks for Predicting a Probability Distribution over Atom Subsets and Chemical Subformulae

Our different enumeration approaches map to potential fragments, represented as either atom subsets or chemical formulae. For basic molecule identification, this often suffices—molecules of radically different structures will have fragments with nonoverlapping peak distributions. However, as molecules get larger and more complex, significant overlap between their spectra can occur, even for molecules without significant structural similarities. Since more information about structure is captured in relative peak intensities, we would like to increase the precision of our barcode spectra by assigning different likelihoods to observing fragments. To do so, we employ graph neural networks (GNNs) as function approximators to learn a feature embedding for every atom in a molecule.^20−25^ Rather than using GNNs directly to learn a molecule embedding or fingerprint that we map to a spectrum, we use them indirectly to learn per-atom features.

The feature embedding stored at each atom represents local information about the atom’s neighborhood and global information about the molecule. The chemical subformulae contains information about which elements are present in a fragment, and how many. Similarly, an atom subset contains more specific information about the atoms that are present in a fragment. The core idea is to combine these two sets of information from the learned per-atom feature embedding and the fragment features to produce a probability distribution over chemical formulae (*FormulaNet*) or atom subsets (*SubsetNet*). Once we have a probability distribution over atom subsets (chemical subformulae), we can directly evaluate what the predicted spectrum would be.

#### Per-Atom Feature Embedding via a Graph Neural Network

**Figure 3:**
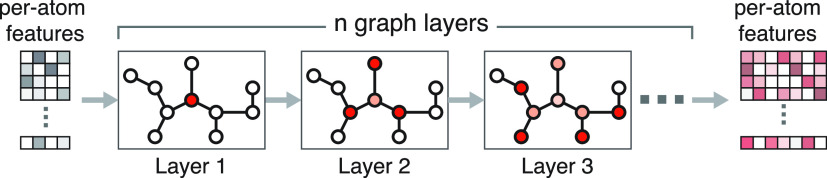
Message-passing graph neural network (GNN). We start off with a vector of features for each atom as our input features for the graph. Each successive layer of the GNN performs an update of each atom’s embedding based on a nonlinear transform of the embeddings of the atoms adjacent to it (hence “message-passing”). After *n* iterations, we generate a new set of embeddings for each atom.

For a molecule graph *M* = (*V*, *E*) with *N*_A_ atoms, we derive *F*_0_ features for each atom (see the Supporting Materials for an exact description of features and network architecture), giving a feature matrix *X*_0_ of shape *N*_A_ × *F*_0_. The bonds between atoms are represented as a symmetric adjacency matrix *A* ∈ {0, 1, 1.5, 2, 3}^*N*_A_×*N*_A_^, where different bond orders are represented by different values. Together, we feed the per-atom feature matrix *X*_0_ and adjacency matrix *A* into a multilayer message-passing graph neural network (GNN) that outputs a per-atom feature embedding  (Figure 3).

#### FormulaNet

The per-atom features *X*_*d*_ can be combined with the atom subset/subformula information in a few ways. The first model we discuss uses only the set of all chemical formulae that arise from a molecule’s fragmentation. Note that the chemical formula enumeration process is simple yet fully exhaustive, combinatorially capturing all possible formula that could arise, even the ones inaccessible via a physical-based fragmentation process.

Our universe of elements is E = {H, C, O, N, F, S, P, Cl}. These eight elements are chosen to ensure nearly full coverage of molecules from PubChem and NIST. Each chemical formula is represented by a count-encoded presentation, an one-dimensional array of non-negative integers representing how many atoms of each element are present . If a molecule generates *f*(*M*) total chemical subformula, then the count-encoded representation our model takes is a two-dimensional array . Within the model, the count-encoded representation is converted into a run-length one-hot encoding of form , where maxelem is chosen to be sufficiently large so as to contain all chemical formulae within the dataset. As an example, the formula CH_3_ may be encoded as [1, 1, 1, 0, 0, 1, 0, 0, 0, 0] where the first five entries correspond to 5 maximum possible H atoms and the last five entries correspond to five maximum possible C atoms. The Supporting Information contains exact details on how this is done.

**Figure 4:**
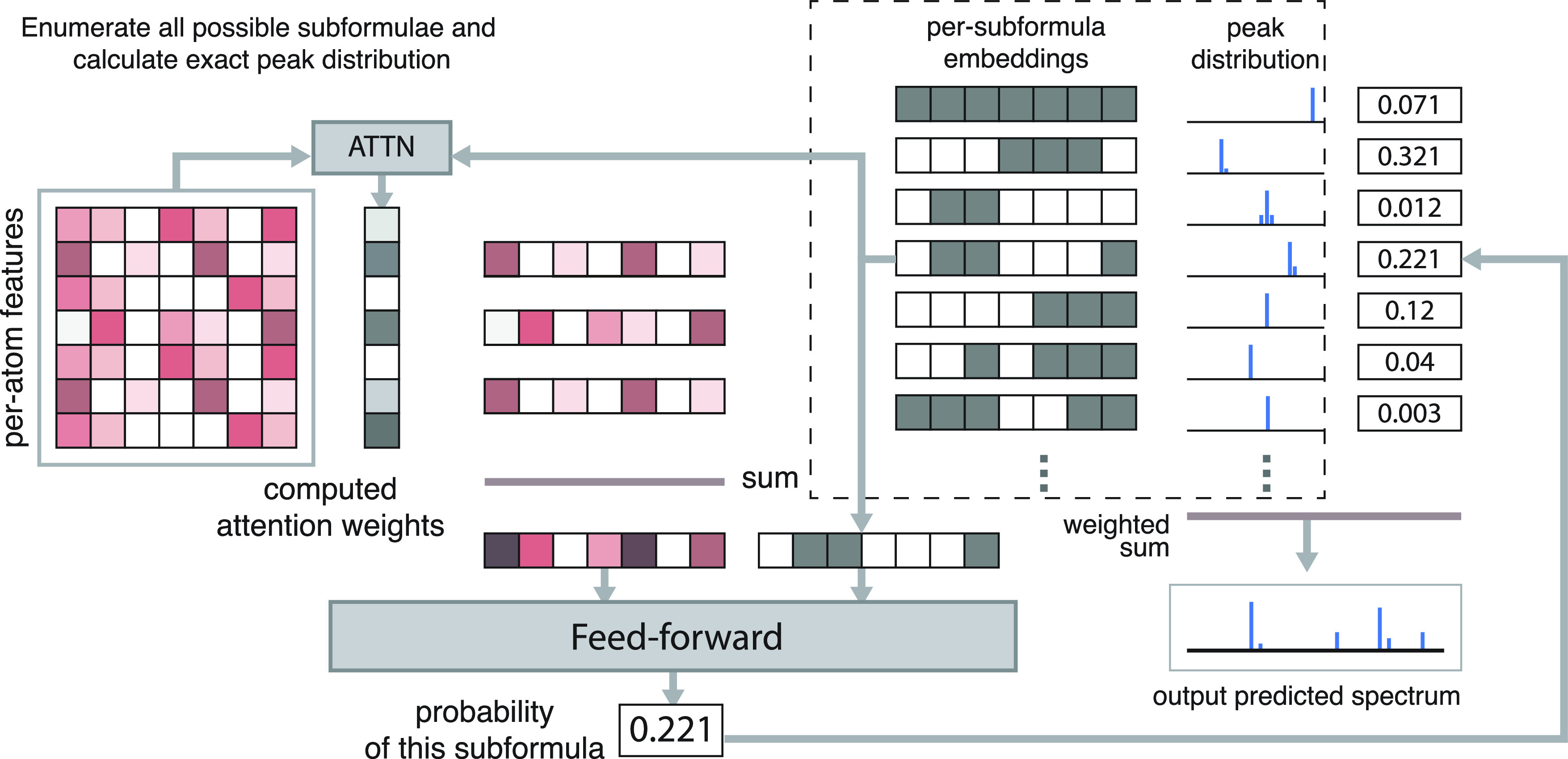
FormulaNet. We compute per-atom feature embeddings using a graph neural network (GNN). We then compute an attention weight for each atom’s embeddings using the attention mechanism described in the text, and use that to perform a weighted sum of those features to produce a subformula-dependent graph embedding. We combine this with the representation of the subformula and (after several feedforward layers) derive a probability that that subformula contributes to the final spectrum.

We then compute an attention operation using the formula embeddings *F*_c_ as key and the per-atom features *X*_*d*_ as query and value. We then concatenate the result with the formula embeddings: [attention(*F*_c_, *X*_*d*_, *X*_*d*_), *F*_c_] and pass this through a MLP to get unnormalized scores *S* for each formula. The unnormalized scores are converted to formula probabilities *p* using a softmax and scaled against weights computed via a linear layer from the per-atom features *X*_*d*_ (Figure 4).

#### SubsetNet

**Figure 5:**
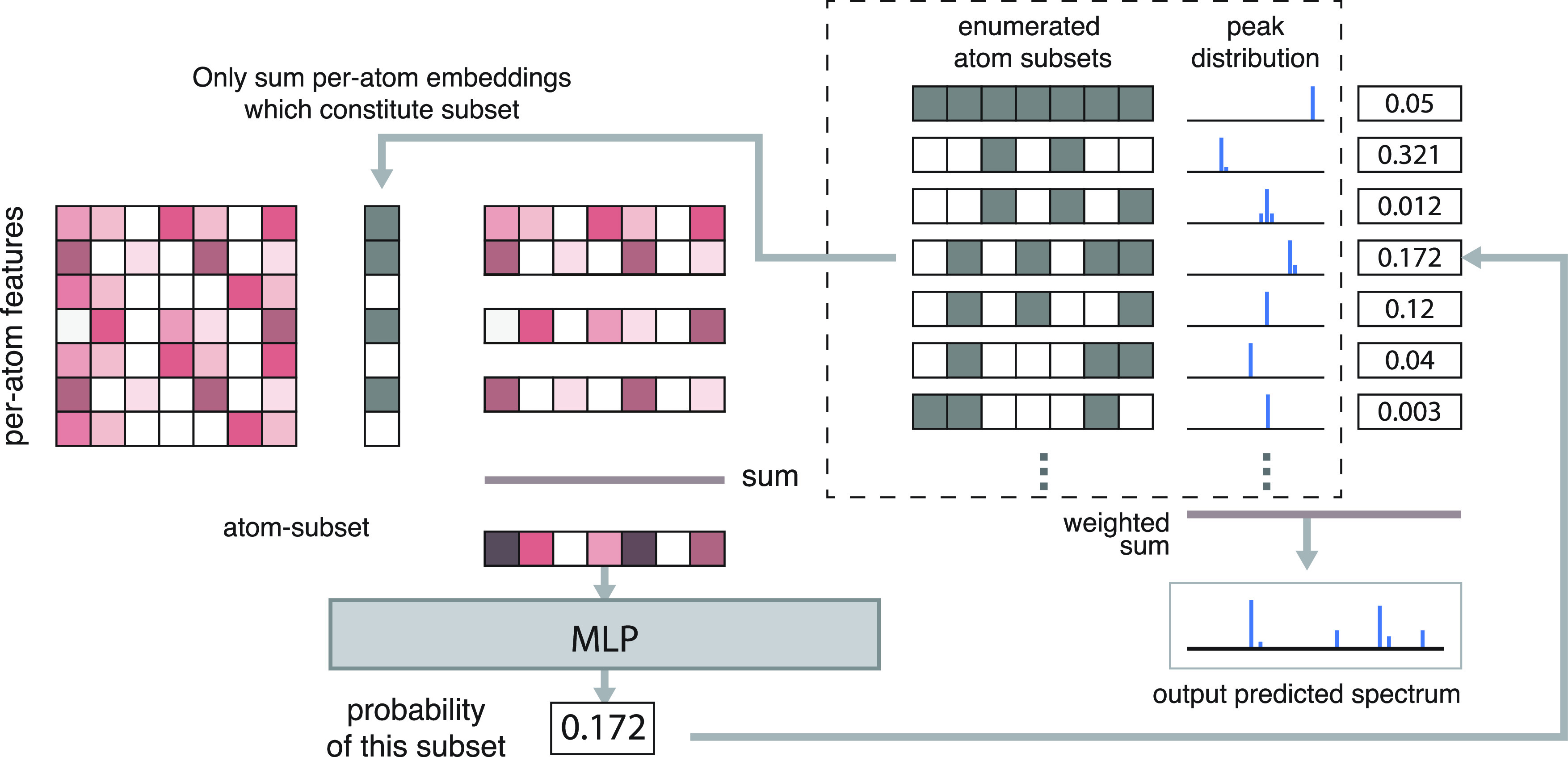
SubsetNet. Like FormulaNet, we use the GNN to generate per-atom feature embeddings. Separately, we generate candidate atom subsets via direct substructure enumeration (bond breaking and rearranging). The per-atom feature embeddings are combined using the atom subsets as “masks” to sum only the embeddings for the atoms present in each subset, generating an embedding for each atom subset. These subset embeddings are then fed into an MLP to generate probabilities for each subset.

The direct fragmentation process generates a set of atom subsets. For a molecule *M* = (*V*, *E*) with *N*_A_ atoms and *N*_S_ unique atom subsets, the subset indicator matrix is a binary matrix of {0, 1}^*N*_S_×*N*_A_^ with 0 indicating the absence and 1 indicating the presence of an atom in a subset. We generate an embedding for each subset by taking the mean of the per-atom embeddings *X*_*d*_ for only the atoms present in each subset. The subset embeddings *X*_*d*+1_ and the run-length *N*-hot encoding of the formula for each subset *F*_r_ are combined and then fed into a MLP to generate probabilities for each subset (Figure 5).

### Observation Model

Both RASSP:FN and RASSP:SN generate probability distributions, the first over unique chemical formulae and the second over atom subsets of the original molecule. Given a formula, we can exactly calculate the observed spectrum, taking into account isotopic variability at natural abundance and mass defect. At integer-Dalton resolution, summing the atomic masses and rounding is sufficient, but using the exact spectral distribution will prove useful for later high-resolution experiments.

We then weight each formula/subset’s mass spectrum according to the model’s output probability and sum all of the observed mass spectra together to obtain one final mass spectrum prediction for the entire molecule.

### Learning Model Parameters from Data

Note that for both models, the input consists of the molecule graph and either (1) a set of possible chemical subformula of the molecule or (2) a set of possible atom subsets of the molecule. The output is a probability distribution over the subformulae or atom subsets. Because the exact mass peak distribution is known for each subformula and subset (Observation Model section), we then exactly compute the mass spectrum at arbitrary resolution. We fit each model using stochastic gradient descent against minibatches of experimentally observed (molecule, spectra) pairs to minimize the L2 error between scaled spectra, where the spectral intensities are scaled by a power. Powers < 1 reduce the importance of outlier peaks, whereas powers > 1 emphasize the importance of outlier peaks.

#### Metrics

Each spectrum is represented as a set of charge-to-mass ratios, intensity tuples (*m*_*k*_, *I*_*k*_). We assume that all measured ions have charge one, and as such, the charge-to-mass ratios may be interpreted directly as masses. Nearly all EI–MS data is obtained at integer-Dalton resolution, i.e., (1.0, *I*_1_), (2.0, *I*_2_), ···. For peaks that do not conform to this specification, such as output peaks from CFM-ID^8^ that specify the exact fragment mass, we transform spectra from a set of discrete peaks to a histogram by binning at integer-Dalton resolution, with bins centered on integer values with unit widths and summing all of the intensities for peaks falling within the same bin. After binning the spectrum, we normalize it to have unit L2 norm.

The key metric for forward model performance is the weighted dot product (eq 1). The weighted dot product scales each mass by a mass power and each intensity by an intensity power. Note that due to the normalization factors on the bottom, this metric is actually weighted cosine similarity and not a proper dot product. Due to the normalization, the values of the weighted dot product (for any *a*, *b*) fall in the range [0, 1].
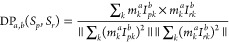
1

Some common values include (*a*, *b*) = (1, 0.5) (regular dot product, DP) and (*a*, *b*) = (3, 0.6) (Stein dot product, SDP).^6^*a* ≥ 1 increases the weight placed on errors at large masses, and *b* < 1 reduces the impact of outlier intensity values. SDP is commonly used in the literature to search and match spectra against spectral databases.^6^

Beyond the dot product (DP) and Stein dot product (SDP), we also track intensity-weighted barcode precision (WP) and intensity-weighted false positive rate (WFPR). These additional metrics, respectively, represent how much of the predicted spectral intensity was in bins also seen in the true spectrum and how much of the predicted spectral intensity was in bins not seen in the true spectrum. For barcode precision, a bin was considered only if the L1-normalized intensity surpassed some cutoff *i*_min_. In this paper, we use *i*_min_ = 0.0001. Top-K precision is also a relevant metric (how many of the top-K peaks in the predicted spectrum are also in the true spectrum). This and further metrics may be found in the Supporting Information.

#### Datasets

The primary dataset used for training both SubsetNet and FormulaNet models was the NIST 2017 Main Library.^9^ After filtering the dataset down to molecules containing only HCONFSPCl atoms, with total atoms ≤ 48, number of unique fragment formulae ≤ 4096 we obtained a dataset of 125 643 molecules. Each molecule was divided into 10 mutually exclusive dataset folds according to the last digit of the CRC32 checksum of the hashed Morgan fingerprint for the molecule. This procedure groups identical molecules in the same dataset fold, acting as an automatic check against repeated rows or molecules in the dataset. We used the first eight folds for training (2–9, 100 438 molecules, nist-train) and the last 2 folds for validation (0 and 1, 25 205 molecules, nist-test).

To compare effectively with CFM-ID,^8^ which provides spectra for evaluation on a small subset of the NIST 2014 Spectral Library, we generate the smallmols-orig dataset from their provided molecule list.^8^ In addition, we pulled molecules from the PubChem Substance database.^10^smallmols-orig was filtered in the same way as the nist17-mainlib (HCONFSPCl atoms, ≤48 atoms, ≤4096 unique fragment formula) and used for evaluation against publicly available parameters for the CFM-ID model^8^ and the NEIMS model.^7^ More information on datasets used is available in the Supporting Information.

The final model with highest SDP and recall at 10 was FormulaNet (see the Supporting Information for exact model parameters). The trained model generalizes to molecules of arbitrary size and fragments, so we evaluated it against the 73.2M PubChem molecules with HCONFSPCl atoms, ≤64 atoms, ≤32 768 max unique fragment formulae, and ≤49 152 max vertex subsets. All of the molecules and spectra are indexed and publicly-available at our website spectroscopy.ai.

The NIST Replicate dataset consists of 63 741 total “replicate” experimental measurements of 23 200 unique molecules. None of these molecules appear in the NIST Main Library. Each molecule was replicated a minimum of two times, with a mean of 2.7 replicates, a median of 2, and a maximum of 24 replicates. This dataset allows us to measure the variability of the experimental process due to stochasticity and inconsistent apparatuses. We use the replicate dataset to estimate the run-to-run variability between measured spectra contributed by varying apparatuses and protocols around the world. This experimental noise provides an upper bound on forward model performance.

## Results

### EI–MS Forward Prediction

**Figure 6:**
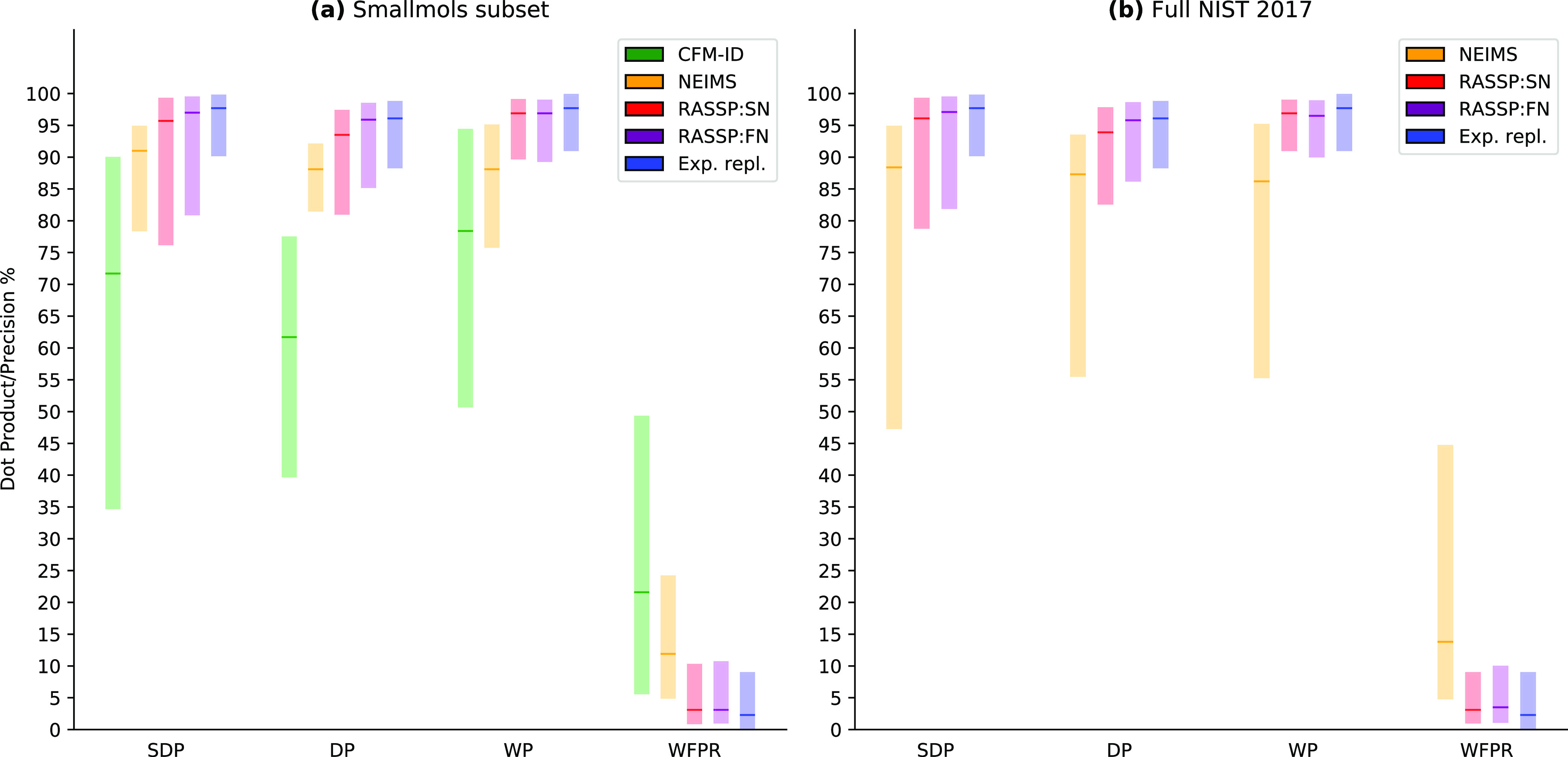
EI–MS prediction performance: the bottom and top of the bars represent the 10th and the 90th percentiles, respectively, with the middle bold tick representing the median (all percentiles evaluated over the dataset specified). (a) Performance of CFM-ID, NEIMS, SubsetNet, and FormulaNet models on molecules from smallmols-orig (a subset of NIST EI–MS data selected in a previous paper^8^). (b) Performance of NEIMS, SubsetNet, and FormulaNet models on nist17-mainlib. Metrics are the Stein dot product (SDP, weighted dot product with (*a*, *b*) = (3, 0.6)), regular dot product (DP, (1, 0.5)), intensity-weighted precision (WP), and intensity-weighted false positive rate (WFPR). “Exp. repl.” refers to experimental replicate variability, estimated by taking the mean metrics over all replicate experiments in nist17-replib and are shown in both (a) and (b) for comparison purposes. They can be viewed as a proxy for experimental variability and as such an “upper limit” to the forward prediction accuracy.

Example spectral predictions are presented in Figure 1, and forward prediction metrics are presented in Figure 6. SubsetNet (RASSP:SN) and FormulaNet (RASSP:FN) were trained for 40 full epochs against a subset of the NIST 2017 EI–MS Spectral Library after selecting for molecules with ≤48 atoms, ≤4096 max unique subformulae, and ≤12 288 subsets (100 438 molecules from nist17-train). A subset of molecules was held out and used as a validation set for tuning hyperparameters and model architectures (nist17-test). Where relevant, RASSP:SN and RASSP:FN refer to the models of each architecture with best performance on this validation set. Where available, performance was also compared against the CFM-ID and NEIMS forward models.^7,8^ NEIMS^7^ was trained from scratch for 100 epochs on nist17-train. CFM-ID spectra for the smallmols subset were derived from the Supporting Data provided by the authors. Full model details, the training process, and code are available in the Supporting Information.

As we can see in Figure 6a, our models show significant improvement in performance over previous physics-based models (CFM-ID), achieving a 95% SDP (out of 100%, actual values are bounded in [0, 1]) on smallmols compared to the CFM-ID 68%. FN and SN outperform NEIMS significantly on both the smallmols dataset and the nist17 datasets. We leverage the nist17 replicate experiments to compute the best possible intra-experimental performance (labeled “Exp. repl.”). Our prediction performance approaches this experimental accuracy, as depicted in Figure 6b. This gives us a sense of the run-to-run and apparatus-to-apparatus variability in the EI–MS process, providing an upper bound on forward model performance.

**Figure 7:**
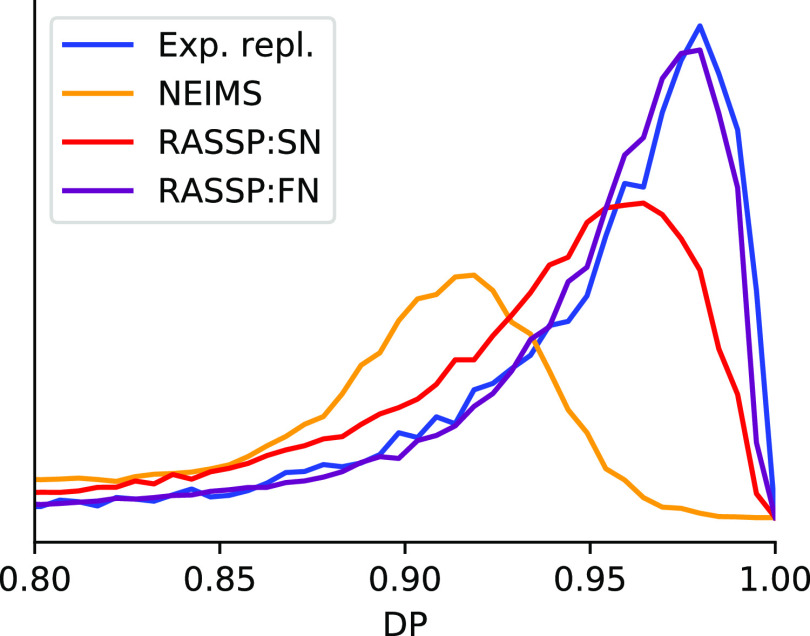
Histogram (probability density function) of prediction dot products DP_1,0.5_. Here, we show the distribution of dot products for all predictions on the NIST Mainlib from the 3 models NEIMS, SubsetNet, and FormulaNet as compared to the distribution of dot products for replicate experiments from NIST Replib (labeled “Exp. repl.”). As forward models improve their accuracy, the distribution should shift to the right. The NIST Replib distribution represents the current limit of prediction performance, accounting for intrinsic experimental variability and differences in experimental setups.

The actual distribution of DP values is depicted in Figure 7. As we can see, the distributions for both SN and FN skew much closer to that of experimental variability than NEIMS. There remains some room for improvement, especially with SN. This indicates how much headroom there might be left to improve upon by improving forward model predictive performance.

### Library Matching

Another validation of the accuracy of our predicted spectra is to use them in a database lookup (library matching) task resembling the common comparison of experimental spectra against spectral databases to identify unknown compounds. We follow the procedure detailed in the NEIMS paper:^7^ we evaluate the performance of an EI–MS forward model using model-inferred spectra to replace a set of molecule, spectra pairs in a spectral database, and then comparing known experimental “replicate” (molecule, spectra) pairs to the database to see whether the true molecule is ranked highly.

We use the NIST 2017 Main and Replicate libraries (nist17-mainlib and nist17-replib, respectively) for this task. The Replicate library consists of replicated experimental measurements and has no overlap with the Main library. To evaluate a given model’s library matching performance, we evaluate it against all molecules in the Replicate library. These spectra are then added to the Main library to form an augmented library that consists of mainlib experimental spectra and replicate model-inferred spectra. We use the Replicate library as a query library, randomly selecting a replicate experimental spectrum for each molecule. Each mol, the spectrum row in the query library is then tested against the augmented library. The max peak in the query spectrum is used to filter the augmented library molecules to ±5 Da, and then, the rows from the augmented library are sorted by decreasing SDP vs the query spectrum. The rank of the matching spectrum is recorded. Some examples of the library matching task are illustrated in Figure 9.

**Figure 8:**
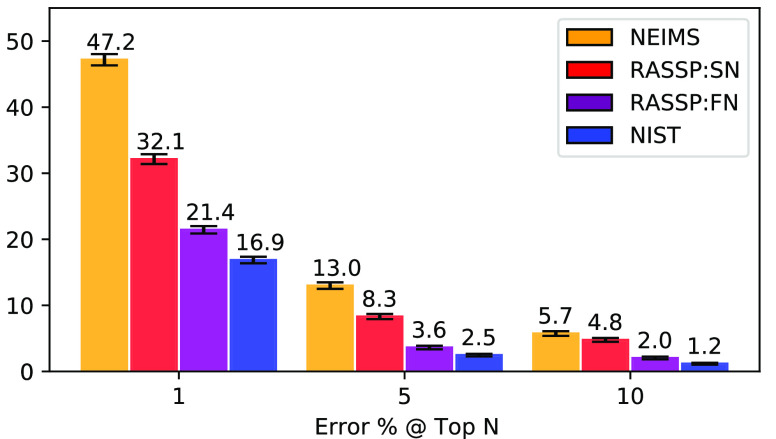
Library matching performance. Comparison of the error rate on the library matching task^7^ over the top 1, 5, and 10 ranked spectra achieved by different model architectures. All graphics display the performance of using NIST replicate spectra as query spectra, indicating the lower bound of error rate, given present EI–MS experimental accuracy. Error bars correspond to 1 – σ variation when estimating the error rate using bootstraps, drawing 20% of the query library randomly without replacement.

As seen in Figure 8, both SN and FN outperform NEIMS in the library matching (database lookup) task they originally detailed.^7^ The error rate at 1 for NIST, at 16.9%, indicates that doing a simple database lookup and taking the top matching molecule gets the wrong match 1 out of every 6 spectra. We improve the error rate at 1 from 1 in 2 spectra (47.2%, NEIMS) to 1 in 4.6 spectra (21.4%, FN). The numbers improve rapidly as the window increases, with the error rate at 10 declining to 1 in 83.3 molecules (1.2%, NIST Ref). FN improves on NEIMS by nearly 3× in this library matching task. Moreover, we note that SN and FN were trained to maximize forward metric performance (SDP), not recall at 10.

**Figure 9:**
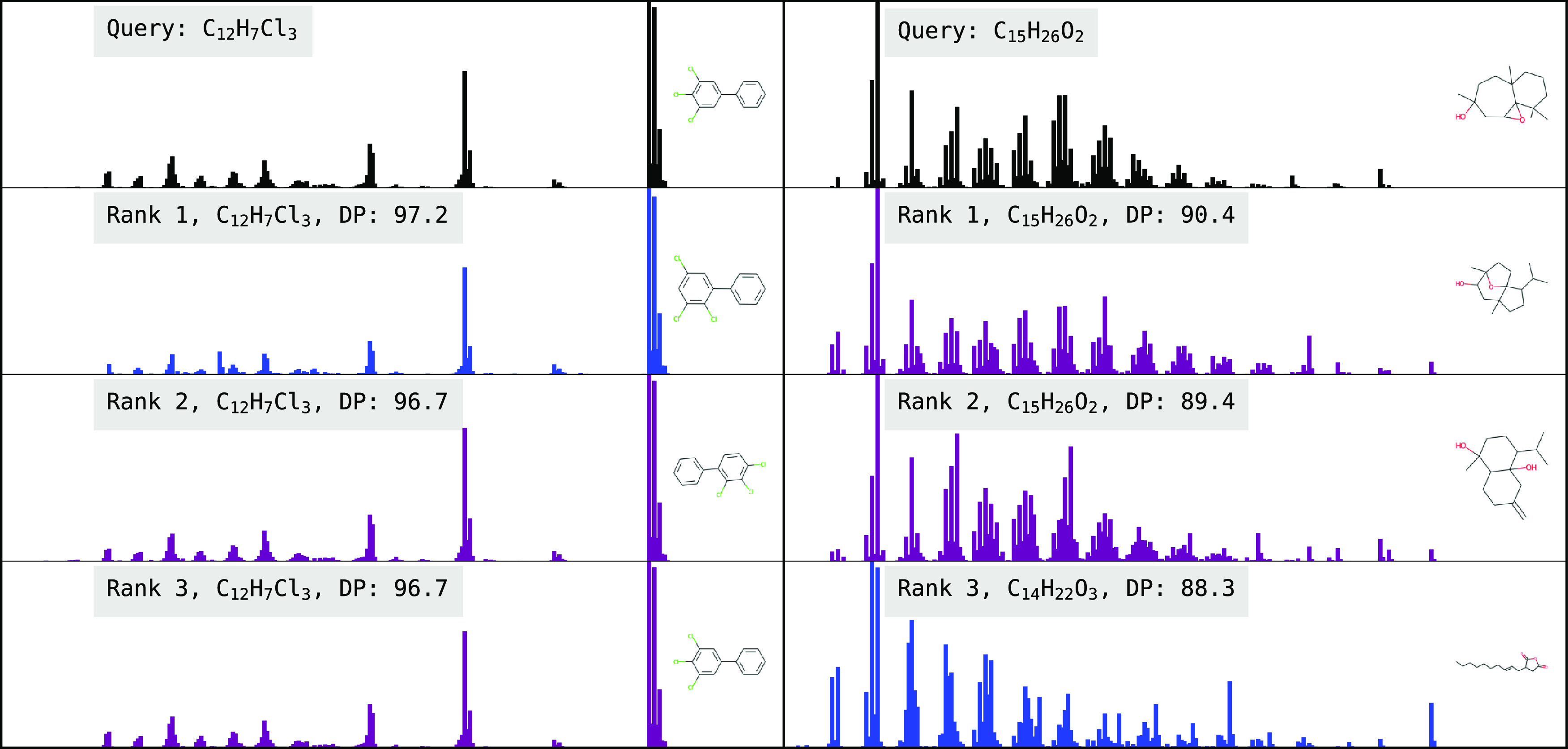
Library matching task. The left and right panels demonstrate two examples of the library matching task. The query spectrum (experimental spectrum from the NIST Replib) is displayed at top in black, and the top 3 ranked spectra from the augmented database (comprised of NIST Mainlib experimental spectra and model-predicted spectra on the NIST Replib) are shown, along with their chemical formulae and the similarity metric (dot product with (1, 0.5)). Blue spectra are experimental spectra from NIST Mainlib and purple spectra are the predicted spectra from the model used in the task. In this figure, predicted spectra are output from the best FormulaNet (FN) model. On the left, we see that the correct match is the spectrum at rank 3. Two molecules with exact formula matches but slightly different structures (hydrogen placements) are ranked higher. On the right, the correct match is ranked outside the top 3, but we can see that two molecules with matching formulae but slightly different structures are ranked at the top.

### Higher-Resolution Data

**Figure 10:**
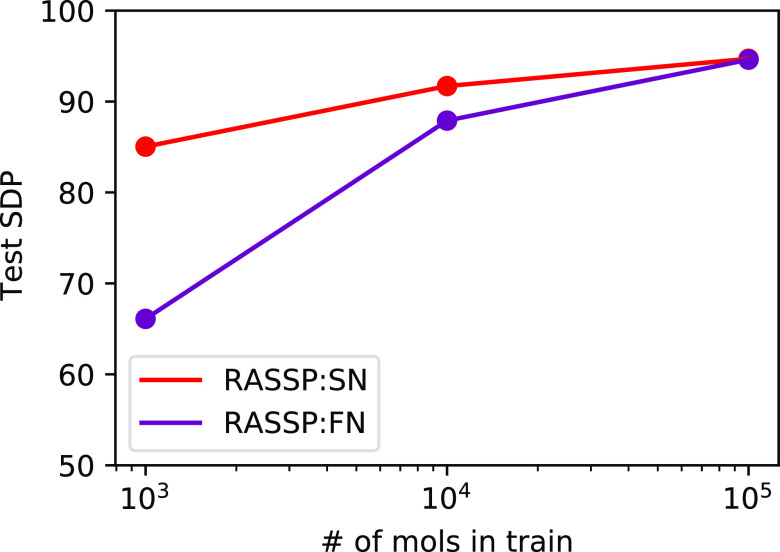
Performance of SubsetNet and FormulaNet with scaling dataset size. As we increase the size of the high-resolution training dataset (synthesized using CFM-ID^8,17,18^ for molecules from PubChem), we see that SN and FN both converge to similar performance. However, their performance diverges dramatically when the dataset is small.

Nearly all computational prediction and database lookups use EI–MS spectra measured at integer-Dalton resolution. Our results detailed here are similar. To test whether either of these models generalizes to higher-resolution data, we trained both SN and FN against a high-resolution synthetic dataset generated using the CFM-ID^8^-provided weights to predict spectra (and their exact peaks) for molecules from PubChem. Rather than binning at the 1 Da resolution, we binned at 0.10 Da resolution. We randomly selected 1000, 10 000, and 100 000 molecules to use as training and held out 10 000 molecules to use as test. The generalization performance of SN and FN is depicted in Figure 10. We see that the performances of SN and FN converge as the dataset size (and molecular diversity) increases, but SN generalizes much better at low-dataset size. Due to the limited availability and expense of collecting high-resolution EI–MS data, this indicates that SN may generalize far better in the low-dataset regime than FN, indicating that the atom subset representation generated by substructure enumeration may be a more natural representation of the mass spectral problem than simply enumerating the formulae. For full details about the generation of the high-resolution synthetic dataset, see the Supporting Information.

### Dependence on Molecular Similarity

Ultimately we are interested in our model’s performance on unseen structures. Machine learning methods learn to recognize patterns in their training data, and thus, care is taken to separate train and test datasets. Fitting of our model is performed exclusively on molecules in our identified training set with test molecules reserved solely for metrics evaluation. In computational spectral prediction, training and evaluating a model on molecules of a particular class or structural motif can lead to inaccurate evaluation of its performance.

To further investigate how our model may generalize to unseen structures, we examine how our model’s predictions on molecules in the test set change depending on how structurally similar those test molecules were to molecules in our training set. Such analysis is key in determining whether a model truly generalizes to structures it has never seen before and can provide further confidence in using its predictions on molecules with no observed spectra.

#### Forward Spectral Prediction Performance

**Figure 11:**
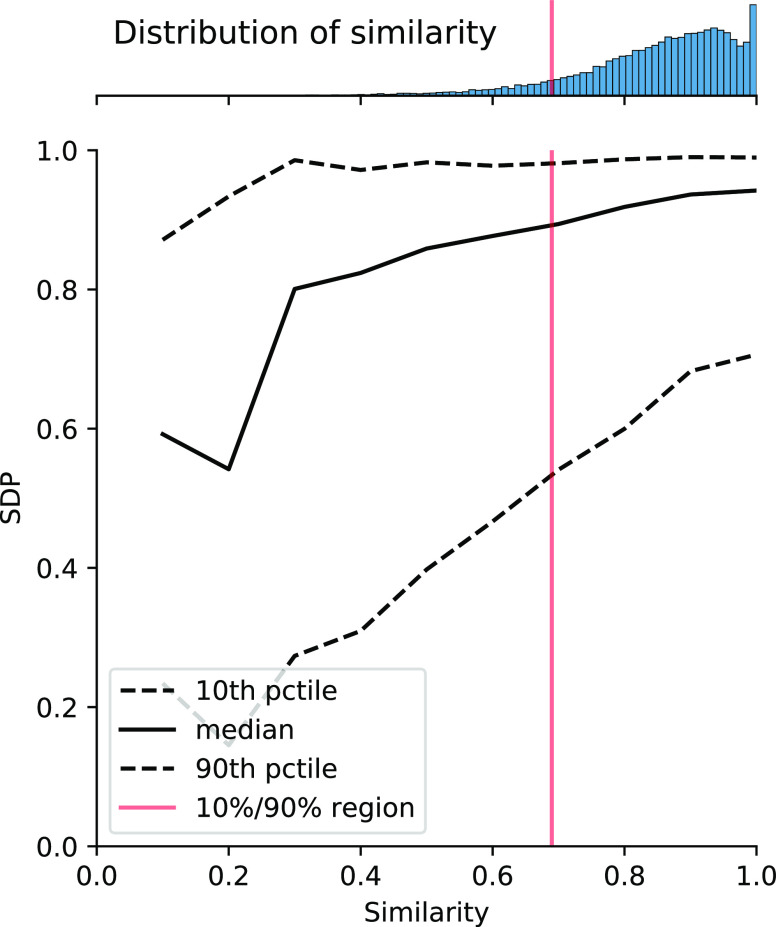
Stein dot product (SDP) vs Tanimoto similarity of our test molecules (*n* = 25 205) to the closest molecule in the training dataset (*n* = 100 438). Results are binned to the nearest decile and the 10%–50% (median)–90% percentiles within each bin are plotted. Additionally, the histogram of the similarities is shown inset above the plot. The vertical red line is the 10th percentile of similarity, plotted at similarity ≈69.0%. Test set molecules (10%) fall below this similarity value, and 90% of test set molecules fall above.

In Figure 11, we present the SDP vs similarity to the closest molecule in the training set for all of the molecules in our test set. We see a clear dependence on similarity—the higher the similarity to the training set, the better the performance. This effect is most pronounced at low similarity levels, where the SDP for the 10% similarity quantile falls to below 20%. Note that 90% of test set molecules have a similarity to the training set over 69.0% (vertical red line).

#### Library Matching Performance

In the library matching task, the NIST Replicate Library we use as the query set features molecules that are not seen in the Main Library. Thus, for each molecule in the Replicate Library, we compute its similarity to the Main Library as the similarity to the closest molecule in the Main Library. We bin the molecules into “low similarity” molecules (*n* = 29 339) and “high similarity” molecules (*n* = 18 771). The cutoff is 90%, below which a molecule is classified as “low similarity”, otherwise “high similarity”. Low-similarity molecules have a mean log 10(rank) of 0.11, whereas high-similarity molecules have a mean log 10(rank) of 0.14. This intuitively makes sense—Replicate library molecules with high structural similarity to Main Library molecules are likely to have similar spectra in the database, and similar spectra can often be hard to distinguish from each other, causing the lookup rank to be higher (worse identification) than molecules with lower similarity. More detailed statistics can be found in the Supporting Information.

### Evaluating the Impact of the Subset Enumeration

**Figure 12:**
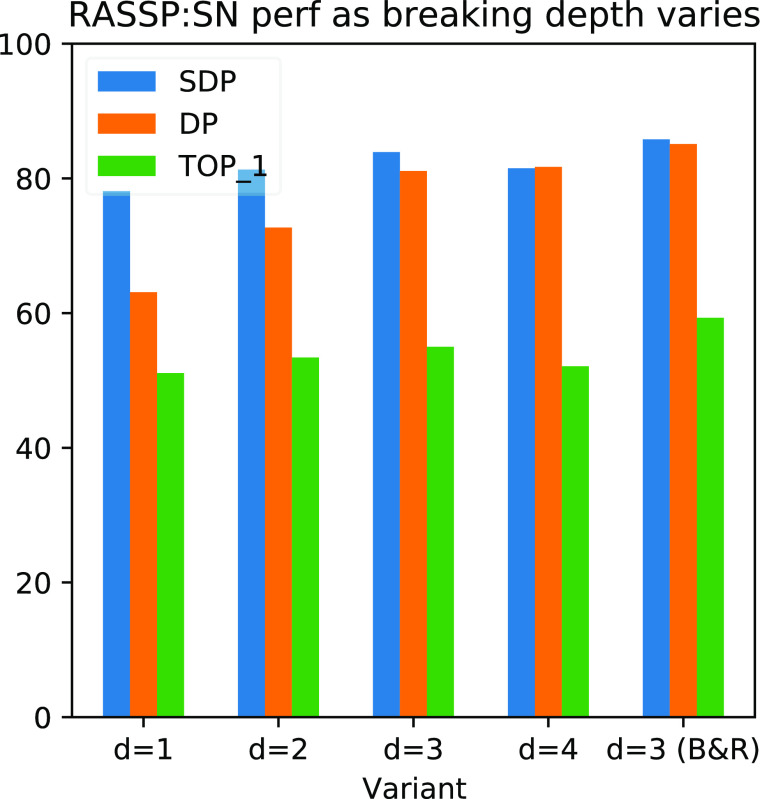
Performance of SubsetNet as depth of bond breaking increases. We fix a SubsetNet architecture and dataset (nist17-mainlib) and vary the depth to which we break bonds, affecting the number of generated substructures and atom subsets. Training is terminated after 1000 epochs and the final performance on the validation set is reported here. We see that as depth increases to *d* = 3, performance increases, but tapers off at *d* = 4. In addition, adding hydrogen rearrangements (B&R) boosts performance over simply doing more bond breaking.

The way we enumerate substructures (here, atom subsets and chemical subformulae) is critical. Chemical subformulae can be completely enumerated without knowledge of the molecule structure, but atom subsets require bond breaking and hydrogen rearrangements. As we increase the depth to which we break bonds, we generate more fragments and should expect monotonically increasing recall and coverage of spectra. In Figure 12 we study the final performance of trained SubsetNets, where all parameters are held constant except for the bond-breaking depth used to generate atom subsets. Each model is trained for 1000 epochs or until the validation SDP no longer increases. The highest-performing checkpoint as measured by validation SDP is selected for final metrics. As we increase the depth to which we break bonds from *d* = 1–3, we see increases in forward similarity (SDP and DP) but a decrease at *d* = 4. The decrease may be due to the way we randomly select a subset of the atom subsets to fit the entire atom subset indicator matrix on GPU. Randomly subsampling the generated atom subsets may throw out important fragments that we no longer consider for weighting and observation later in the pipeline. In this paper, we only focus on the subsets achievable by bond breaking out to depth 3. Notice that if we add hydrogen rearrangements (“*d* = 3 B&R”), we continue to see improvement in performance. This indicates that further improvements in the recall and physical plausibility of the generated subsets are likely to boost performance, in addition to increasing the number of atom subsets considered for observation.

## Discussion

Previous efforts to learn machine learning models from mass spectral data have focused on better rule-based fragment enumeration schemes or used machine learning (graph neural networks, transformers) to directly predict spectra from molecule embeddings (SMILES strings, fingerprint hashes, etc.). Comprehensive substructure enumeration methods tend to have high recall at the cost of low precision, whereas machine learning tends to help recover that precision. In this work, we combine a physically plausible substructure enumeration process with GNNs, demonstrating that this fusion outperforms all previous models. We present *SubsetNet* and *FormulaNet*, two models for predicting EI–MS spectra. *FormulaNet* significantly outperforms all previous methods of EI–MS spectral prediction, achieving an average SDP of 92.9% and DP of 93.5% over the largest publicly available database of EI–MS spectra. In addition, our predicted spectra may be evaluated indirectly by utilizing them in a library matching (database lookup) task. Here, we also outperform previous methods, achieving a recall at 10 of 98.0%. *SubsetNet* does much better at generalization in the low-data regime by leveraging more fine-grained information about substructures. Such performance approaches the limits of experimental data (see Figure 7). We generate EI–MS spectra predictions for 73.2M molecules from PubChem and make them freely available.

All computational approaches to predicting EI–MS spectra are fundamentally limited by the available data. The largest publicly available spectral library to date is still the NIST Mass Spectral Library.^9^ Experimentalists from around the world are free to contribute EI–MS spectra measured at 1 Da resolution to the library. As higher-resolution tandem MS/MS machines come online, spectral databases will increasingly consist of heterogeneous data, mixing experimental spectra measured at many different resolution scales. Importantly, because RASSP predicts a probability distribution over fragments with known exact mass peak distributions, it can be used to predict spectra at arbitrary resolutions by simply changing how we bin the binning of predicted probabilities. As such, our approach is the first approach that can be used to leverage data from multiple sources, thanks to the ability to train against high and low-resolution data simultaneously. It is common to use some form of dot products or cosine similarity as a spectral similarity metric for measuring forward spectral prediction performance and library matching. However, in higher-resolution tandem MS/MS, the false positive rate may be even more critical. Future work would investigate the importance of different metrics in measuring spectral prediction performance and integrating supervision from both higher-resolution EI–MS spectral data and other types of metadata, such as ionization energy and experimental apparatus.

Each of the modules (subset and subformula enumeration vs machine learning model for the fragments) can be improved independently. For computational ease, our enumeration process generates fragments by breaking up to and including three bonds and also includes all possible hydrogen rearrangements. However, there are more exotic fragmentation schemes that we have ignored, and their inclusion could potentially improve the recall of the generated fragments. The graph neural networks we use only consider the atoms and do not take into account any information about the bonds, other than their bond order. These models may be improved by incorporating edge information and making changes to the model architecture, such as a novel bipartite atom-bond message-passing scheme or other improvements. Together, future improvements may enhance both the recall and the precision of our forward model.

An accurate in silico forward model for predicting EI–MS spectra can be applied to library search and compound identification. Running similarity search over spectral databases using repeated spectral measurements obtained from NIST Replib achieves an error rate of 1% at 10 using DP_1,0.5_, which sets the lower bound on library matching accuracy, given current EI–MS hardware. By augmenting existing spectral databases with in silico spectral predictions from our forward model, we can massively increase the number of molecule candidates considered, potentially increasing the ability for scientists to discover novel and rare compounds. However, the search problem quickly becomes computationally challenging as the number of molecules increases. A typical query over the 300K molecules in NIST Mainlib takes about 100 ms. To improve the computational efficiency of the library matching/database search task, we can use more efficient similarity metrics, approximate computations, and dimensionality reduction via approaches like nearest-neighbor hashing or locality-sensitive hashing. Recent work has already demonstrated that deep learning-based similarity measures can dramatically improve accuracy over simpler cosine similarity measures in database lookup tasks.^26,27^

In the long-term, we expect computational spectral prediction to enable novel applications. For example, computationally obtained spectra may be used to augment metabolomics studies by enabling researchers to automatically match spectra to molecules that have never been experimentally studied. Future work could use a good computational forward model for EI–MS to generate large amounts of training data that could then be used as supervision for an inverse model to further automate this and other types of molecular identification problems. The runtime of these forward models may be improved by further algorithmic improvements to the substructure generation step and the machine learning models.

## Data Availability

The code for this work can be found at github.com/thejonaslab/rassp-public. Predicted spectra for the 70M+ small molecules in PubChem can be found at spectroscopy.ai.

## Supporting Information Available

Pretrained model weights, source code, and additional supporting information detailing datasets used and other details; spectra for the 73.2M PubChem molecules (PDF)
