# Supplementary material for: Rapid Approximate Subset-Based Spectra Prediction for Electron Ionization–Mass Spectrometry
Source: Anal Chem. 2023 Jan 25;95(5):2653–63. doi: 10.1021/acs.analchem.2c02093 (PMC9909676; doi:10.1021/acs.analchem.2c02093)
Supplement: Supplementary file 1 — ac2c02093_si_001.pdf [file ac2c02093_si_001.pdf]

Supplementary Information for Rapid Approximate Subset-based Spectra Prediction for Electron-ionization Mass Spectrometry

Richard Licheng Zhu[†] and Eric Jonas[*,‡]

†University of Chicago, Committee on Computational and Applied Mathematics

‡University of Chicago, Department of Computer Science

E-mail: ericj@uchicago.edu

Data

Preprocessing

All spectra in the NIST 2017 dataset was extracted from the installed software provided upon purchase.[1] After scraping and cleaning (including filtering for only the molecules containing HCONFSPCl atoms) we are left with 241,028 molecule-spectra experiments across 237,189 unique molecules (de-duped using INCHI keys) in `nist17-mainlib`, and 63,741 molecule-spectra experiments across 23,200 unique molecules (de-duped using INCHI keys) in `nist17-replib`. Using INCHI keys as unique identifiers for molecules (hash collisions are possible but extremely rare), we confirm that there are zero molecules common to both `nist17-mainlib` and `nist17-replib`. This is because `nist17-mainlib` is the "Main" library, and `nist17-replib` (the "Replicate" library) is a collection of spectral experiments for molecules that were replicated at least 2 or more times. This database is not used during training and is only used during the library matching task. For other datasets (see Table 1),

we report the number of rows, corresponding to the number of total molecule-spectra experiments. More complete dataset information, including lists of molecules and their metadata, will be made available upon request.

To keep the training runtime at an acceptable level, we filter the training set based on the maximum observed peak mass, the max number of atoms, and the max number of unique fragment formula. In this paper, we train and evaluate against molecules with all mass peaks $\leq$ 511 Daltons, $\leq$ 48 atoms and $\leq$ 4096 max unique fragment formula. However, the way that the models are structured allow us to perform inference against molecules of arbitrary size. We do so when running inference against `pubchem-pred`.

As mentioned in the Main Text, we subdivide our `nist17-mainlib` dataset into train (41.7% of `nist17-mainlib`) / test (10.4% of `nist17-mainlib`) splits by computing the CRC32 checksum of the `morgan4` molecular fingerprint for each molecule and subdividing into splits based on the last digit of the value. This minimizes the chance that exactly identical molecules-spectra experiments or overly-similar molecules are placed into both the training and test sets. Hashed fingerprints ending in $[0, 1]$ were used as the test split, and all others were used as the train split. The function we used to compute the CRC32 checksum of the fingerprint is `morgan4_crc32`, available in `rassp.util` in our released code.

Resolution

All spectra in NIST 2017 are reported at integer Dalton resolution. For downstream training and evaluation, we represent spectra as vectors $s \in \mathbb{R}^{512}$, with bin i containing the observed intensity at charge-to-mass ratio i. For example, bin 1 contains the intensity for $m/z = 1$, bin 2 contains the intensity for $m/z = 2$, and so on. In this paper we do not consider fragments ionized to charge $z > 1$, so the spectra may be directly read off as intensities for a given mass value $m/z = m$. We refer to charge/mass ratio and mass interchangeably in the paper.

Synthetic high-resolution data

In Section 4.3, we discuss performance of SN and FN against a high-resolution synthetic dataset generated by running CFM-ID against molecules from PubChem. We randomly sample 110,000 molecules (100,000 used for training and 10,000 used as a held-out eval set) from PubChem that contain only HCONFSPCl atoms, ≤ 48 atoms, max fragment formula ≤ 4096, and molecular weight ≤ 512. We then run CFM-ID against these molecules using the EI-MS model weights and default configuration as provided by the CFM-ID authors.[2] CFM-ID outputs a list of fragments (in `smiles` form) and the corresponding prediction intensities. We use these results as synthetic high-resolution data, because the fragments have known exact mass and can be binned at arbitrary resolution.

Using the synthetic data, we then construct a dataset by binning at $\Delta m = 0.050$ Dalton resolution. Because we consider molecules with weight up to 512, the dataset contains spectra with $512/\Delta m = 10240$ bins.

We then train SN and FN from scratch against this dataset, varying the number of molecules over 3 orders of magnitude via subsampling: 1k, 10k, and 100k. The metrics reported in Fig. 10 are obtained by taking the best-performing model on each run and evaluating it against the held-out test set of 10k molecules, also binned at $\Delta m = 0.050$ Dalton resolution. Due to the difference in binning between the 0.050 Dalton resolution experiments and the 1 Dalton resolution experiments, test SDPs and other metrics are not directly comparable. However, the relative comparisons between models as we increase the size of the training set are meaningful.

Table 1: Datasets referenced in this paper.

Name	# Mols	Source	Mean # atoms	Max # atoms	Max unique formula	Max weight
`smallmols-orig`	17,322	NIST 2014[1,3]	25.4	87	151,700	772.1
`smallmols-filtered`	13,281	NIST 2014[1,3]	24.0	48	4,095	504.0
`nist17-mainlib`	241,028	NIST 2017[1]	42.3	255	3,427,050	1,674.8
`nist17-replib`	63,741	NIST 2017[1]	30.1	173	823,680	967.0
`nist17-train`	100,438 (41.7%)	NIST 2017[1]	30.1	48	4,096	509.7
`nist17-test`	25,205 (10.4%)	NIST 2017[1]	30.0	48	4,096	510.0
`pubchem-clean`	90,844,616	PubChem DB[4]	47.7	128	43,868,720	2,046.2
`pubchem-pred`	73,198,384	PubChem DB[4]	-	-	-	-
`pubchem-clean-filtered`	27,960,210	PubChem DB[4]	33.7	48	4,096	512.0

SubsetNet and FormulaNet in detail

Input featurization

Suppose $X \in N_A \times D$ to be our feature matrix for a molecule of N_A atoms and D per-atom features. Using the features listed in Table 2, we have $D = 45$ in this paper.

Table 2: Features used for each atom

Feature name	Dimensions
Atomic number (integer)	1
Atomic number (one-hot)	8
Valence (integer)	1
Total valence (one-hot)	6
Aromatic (boolean)	1
Hybridization (one-hot)	8
Formal charge (one-hot)	3
Default valence (one-hot)	6
Ring size (one-hot)	5
Total hydrogens (one-hot)	6
Total dimensions	45

- Atomic number (integer)
- Atomic number (one-hot)
- Total valence (integer)

- Is aromatic (boolean)
- Hybridization (one-hot)
- Formal charge (one-hot)
- Covalent radius (float)
- van der Waals radius (float)
- Default valence (one-hot)
- Total hydrogens (one-hot)

In addition, we also generate the symmetric adjacency matrix which contains bond order information $A \in \{0,1\}^{N_A \times N_A \times 4}$, storing 1 in bin 1 for a single bond, bin 2 for a hybridized bond, bin 3 for a double bond, and bin 4 for a triple bond.

Our featurization pipeline is common to both SubsetNet and FormulaNet, and converts the molecule into a tuple (X, A).

Model details

Graph neural networks for computing molecule and atom embeddings

We cite some useful references for understanding and utilizing GNNs for this and related problems.[5,6]

The first phase of SubsetNet and FormulaNet are GNNs that ingest the per-atom features and the adjacency matrix (X_0, A) and outputs per-atom features/embeddings X_L. Specifically, the GNN is a mapping $f : \mathbb{R}^{N_A \times D_0}, \{0,1\}^{N_A \times N_A \times 4} \rightarrow \mathbb{R}^{N_A \times D_L}$.

SubsetNet. The layers are as follows:

- Batch normalization
- 16 layers of message-passing graph convolutional layers

- 512 × 512 Weight matrix multiply (first layer converts from the input feature dimension $D = 45$ to 512)
- Adjacency matrix masking
- Sum with bias
- LeakyRELU
- Residual sum with the previous layer's output
- Instance normalization - `Batchnorm1d`

The output is a matrix of per-atom feature vectors $X_L \in \mathbb{R}^{N_A \times D_L}$. In our case we set N_A to be a maximum of 64 atoms and $D_L = 512$.

FormulaNet. The first phase of FormulaNet, like SubsetNet, is a GNN. Featurization proceeds as before, and the GNN layers are as follows:

- Batch
- 16 layers of message-passing graph convolutional layers
  - 512 × 512 Weight matrix multiply (first layer converts from the input feature dimension $D = 45$ to 512)
  - Adjacency matrix masking
  - Sum with bias
  - LeakyRELU
  - Residual sum with the previous layer's output
  - Layer normalization - `LayerNorm1d`

The main difference between SubsetNet and FormulaNet's GNN component is the normalization used within each layer.

Parametrizing a probability distribution over the subformula and subsets

In the previous phase, we took as input per-atom feature vectors $X_0 \in \mathbb{R}^{N_A \times D_0}$ and output per-atom embeddings $X_L \in \mathbb{R}^{N_A \times D_L}$. We combine these per-atom embeddings with a separately-constructed enumerations over the possible fragments to produce a probability distribution over the fragments.

SubsetNet. In SubsetNet, the relevant fragments are represented as atom subsets.

The atom subsets are obtained via a direct fragmentation and subset enumeration procedure wherein we recursively break all the bonds out to a given breaking depth $d = 3$, compute the resulting connected components, and throw away information about the edges and retain only the atoms that were present in connected components together as atom subsets. Each atom subset is stored as a vector $s_i \in \{0, 1\}^{N_A}$ with 1 if the corresponding atom was present in the subset, and 0 if not.

The per-atom embeddings from the first phase X_L is then combined with the atom subsets (obtained via direct fragmentation and subset enumeration) to generate per-subset embeddings. Let the atom subset indicator matrix be $S \in \{0, 1\}^{N_S \times N_A}$. The matrix multiplication SX_L gives us a matrix of per-subset embeddings $X_S \in \mathbb{R}^{N_S \times D_L}$, which corresponds to doing a linear combination of the per-atom embeddings for only the atoms present in each subset.

In addition, for each subset we also take its chemical formula and generate a cumulative one-hot binary feature vector. Since we restrict to molecules containing only HCONFSPCl atoms (8 unique elements), we require constraints on the maximum number of allowed atoms for each element. The maximum allowed elements for each element in HCONFSPCl respectively was $[50, 46, 30, 30, 30, 30, 30, 30]$. The corresponding embedding size for any single formula is the sum of the max allowed elements, here 276. Hence, we have the per-subset embeddings $X_S \in \mathbb{R}^{N_S \times D_L}$ and the per-subset chemical formula embeddings $X_{SF} \in \mathbb{R}^{N_S \times 276}$.

The second phase combines the per-subset embeddings X_S and the per-subset chemical formula embeddings X_{SF} via a fully-connected layer, and then additionally pass it through two more fully-connected layers to reduce the per-subset embeddings down to per-subset

logit scores, which are then converted into subset probabilities via softmax.

FormulaNet. In FormulaNet, the relevant fragments are represented as chemical formula. This is essentially taking the atom subset information from above, and taking a quotient operation over the subsets where we identify all subsets that have the same chemical formula as equivalent.

We generate the set of subformulae for a given molecule. As before in SubsetNet, we produce a cumulative one-hot binary feature vector for each subformula.

The formula embeddings and per-atom embeddings X_L from the first phase are then mapped into the same space and an attention operation is taken, amounting to a pairwise comparison between all formula and all atom embeddings. The resulting similarities are converted by softmax into values between 0 and 1, and then used to scale and reduce the per-atom embeddings down to a per-subformula embedding.

Like SubsetNet, the next phase combines the per-subformula embeddings with the per-formula one-hot embeddings using a `GRUCell`. The output is passed through three fully-connected layers (each containing 128 units) to get a per-formula logit score, just as SubsetNet combines the per-subset embeddings with the per-subset chemical formula.

Further details for model implementation are available in our provided code.

Hyperparameters

The loss function used was a simple MSE loss against the square root of spectral intensities. Scaling the intensities by a power of 0.5 in the loss function was intended to de-emphasize outlier intensities. Both models were trained to convergence using the Adam optimizer with learning rate 0.0002.

Training

Both models were trained to convergence after 20 passes over the full `nist17-train` dataset, which took 100 hours on a workstation with 2 Intel(R) Xeon(R) Gold 6130 CPU @ 2.10GHz

CPUs and 2 NVIDIA RTX 2080 Ti GPUs. Inference on small molecules with ≤ 48 atoms and ≤ 4096 max formula on the same workstation achieved an average of 16 molecules per second. All training and inference was performed using 32 threads and a single GPU.

Reproducing results

In our publicly-released code, we provide the model code and configuration files used when producing our results. The NIST dataset is proprietary and cannot be released by us. Instead, we provide the first 100 molecules from the `smallmols-orig` dataset.[3] The INCHI and SMILES strings are provided, in addition to high-res predicted spectra obtained by running the publicly-available CFM-ID EI-MS model.[3] We provide a script that trains a model against this dataset, performs basic forward inference, and computes metrics for the forward prediction task and the library matching task.

Pretrained model weights, including the best FormulaNet and SubsetNet models we trained, are not included with the code package due to size constraints but are publicly-available at `https://people.cs.uchicago.edu/~ericj/rassp/`. Instructions for use are included in the code package `README.md`.

Comparisons to other models

CFM-ID[3]

CFM-ID provided the exact smiles strings corresponding to the smallmols dataset. In order to get the most favorable comparison for CFM-ID, we used the provided spectra (which performed better than the spectra output by the model using the weights provided) as our benchmark in Fig. 6. However, due to lack of coverage of our dataset, we used the provided EI-MS weights and the default configuration to generate spectra from PubChem molecules for the synthetic dataset employed in producing Fig. 10.

NEIMS[7]

We retrained the NEIMS model on the same `nist17-train` dataset. Note that the NEIMS code accepts `.tfrecord` format only. In addition, the code expects spectra to be normalized to have a maximum magnitude of 999 (as detailed in the original paper).[7] We did not use the provided model weights due to their training set containing molecules from both our train and test sets. There is no guarantee that we trained the model optimally, however we did train for a much longer period (100 epochs or passes over our NIST 2017 training set) with the default hyperparameters to ensure that our comparison would be as favorable to the original work as possible.

Runtime

Forward model runtime information is detailed in Table 3.

CFM-ID numbers and NEIMS numbers are pulled from the reported numbers in the original papers.

All training, inference, and benchmarks were performed on a server with 1 Intel(R) Xeon(R) Gold 6130 CPU @ 2.10GHz CPU and 1 NVIDIA RTX 2080 Ti GPUs. Inference runtimes were computed using 16 PyTorch CPU workers for loading data.

Table 3: Forward model runtime

Model name	Runtime (ms) per mol	Mols per second
CFM-ID	300,000[3]	0.0033
NEIMS	5[7]	200
SubsetNet	53	19
FormulaNet	23	44

PubChem inference

We take our best-performing FormulaNet model and evaluate it on `pubchem-pred`, containing 73.2M small molecules from the PubChem database. All molecules and predicted spectra in

`pubchem-pred` will be made available at our public website `spectroscopy.ai`.

Analysis of molecular similarity vs performance

All mentions of molecular similarity refers to the Tanimoto similarity (AKA Jaccard similarity or the ratio of Intersection over Union), defined on two binary arrays as:

$$\text{TanimotoSimilarity}[\vec{f}, \vec{g}] = \frac{\vec{f} \& \vec{g}}{\vec{f} || \vec{g}}$$

where the & operator represents a bitwise-AND operation and the || operator represents a bitwise-OR operation. This similarity measure is a real number in $[0, 1]$.

In these studies, we used the default RDKit fingerprint for molecules (2048-dimension binary bitvector).[8]

Forward spectral prediction performance and similarity

For every molecule in our test set ($n = 25205$), we find its nearest neighbor in the training set ($n = 100438$) as measured by similarity discussed above. We present the scatter plot of SDP (Y-axis) scattered against the similarity to training (X-axis) in Fig. 1 below.

Figure 11 (Main Text) is the same data, but additionally binned for clarity. We bin the similarity in deciles (round to the nearest 10%) and compute the 10%-50%-90% percentile values within each bin. We present the number of molecules in each similarity bin of Figure 11 in Table 4. Note that as the similarity decreases, we have fewer molecules in each bin. The values in lower bins are expected to be more noisy for this reason.

Library matching performance and similarity

For every molecule in the NIST Replicate Library ($n = 63741$) we find its nearest neighbor in the NIST Main Library ($n = 241028$) as measured by similarity discussed above.

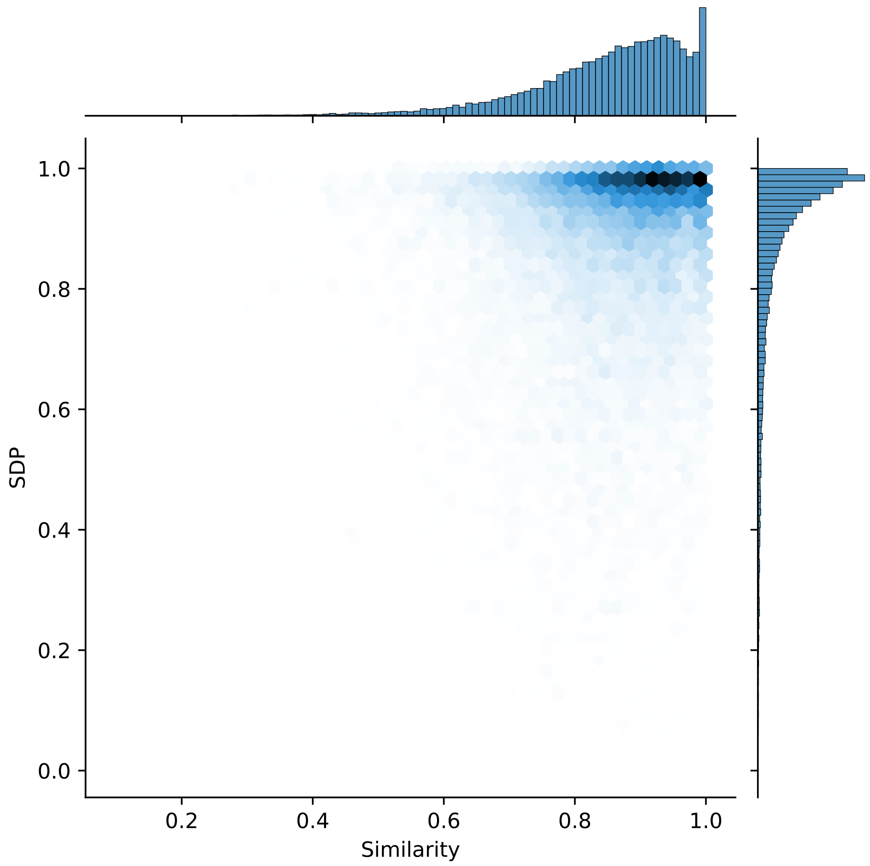

Figure 1: SDP vs similarity hex jointplot

Because matching rank is a heavily skewed value that ranges over several orders of magnitude (the most common matching rank is < 10, but matching rank can often reach 100 or 1000), we do not report 10%-50%-90% percentiles but rather compute the mean in log-space. We use logarithms to base 10.

We present the scatter plot of log10(rank) (Y-axis) scattered against similarity (X-axis) in Fig. 2.

Unlike the forward spectral prediction analysis, we only bin into "low similarity" $< 90\%$ and "high similarity" $\geq 90\%$ molecules here, and compute the mean log10(rank) over each bin. Percentiles make little sense for this data because a majority of molecules have a rank of 1 (log10 rank of 0). The low similarity molecules ($n = 29339$) had a mean log10 rank of 0.110 and the high similarity molecules ($n = 18771$) had a mean log10 rank of 0.135.

Table 4: Number of molecules in each similarity bin for Main Text Figure 11

Decile	n
0%	0
10%	5
20%	25
30%	64
40%	176
50%	440
60%	1146
70%	2756
80%	6550
90%	10117
100%	5188

Additional statistical analysis

We would like to understand how our reported performance metrics (SDP and others for forward spectral prediction, matching rank for library matching / database lookup) vary as our models are trained on different subsets of the data. To do so, we split our dataset into 5 cross-validation splits, and trained 5 different FormulaNet models to 1000 epochs using each split (choose 4 for training, hold 1 out for testing).

For the forward performance, the standard-deviation of headline (the value we report in the abstract) mean SDP (evaluated on the held-out test set, which changes from training run to training run) we see on the order of 0.10%. At the level of individual molecules, we get an average run-to-run std-dev in SDP of 2.1% (over 5 runs).

For the library matching task, we looked at the dispersion in rankings between the 5 models. Because each model is trained on a different subset of data (80% is selected and 20% is held out), there is likely to be gaps where a certain model will fail to rank the query molecule highly. We see this borne out in practice. 91% of the time all 5 models will rank the query molecule in the top 10 molecules and achieve a median rank dispersion (the max delta between the highest rank and the lowest rank achieved by any of the five models) of 0.0 and an average rank dispersion of 13.6. The other 9% of the time, we see a median rank

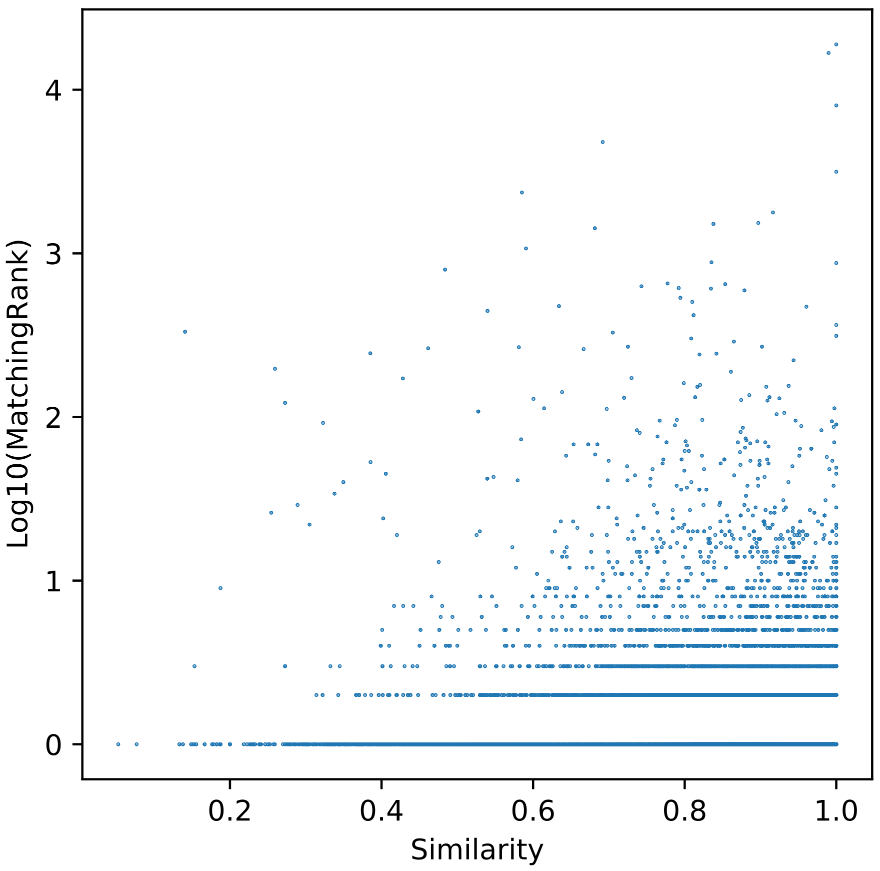

Figure 2: Log10(MatchingRank) vs similarity scatter plot

dispersion of 24 and an average rank dispersion of 152.1, suggesting that when a model does fail, it fails spectacularly in comparison to the others.

This suggests that accumulating more data on diverse molecular structures is key in achieving the best possible performance on both tasks.

Discussion

Glucose example

Given a molecular graph $G = (V, E)$ where the vertexes correspond to atoms and the edges correspond to bonds, our subset enumeration process outputs a list of atom subsets. These subsets are not randomly generated by choosing a subset of the atoms, but are instead

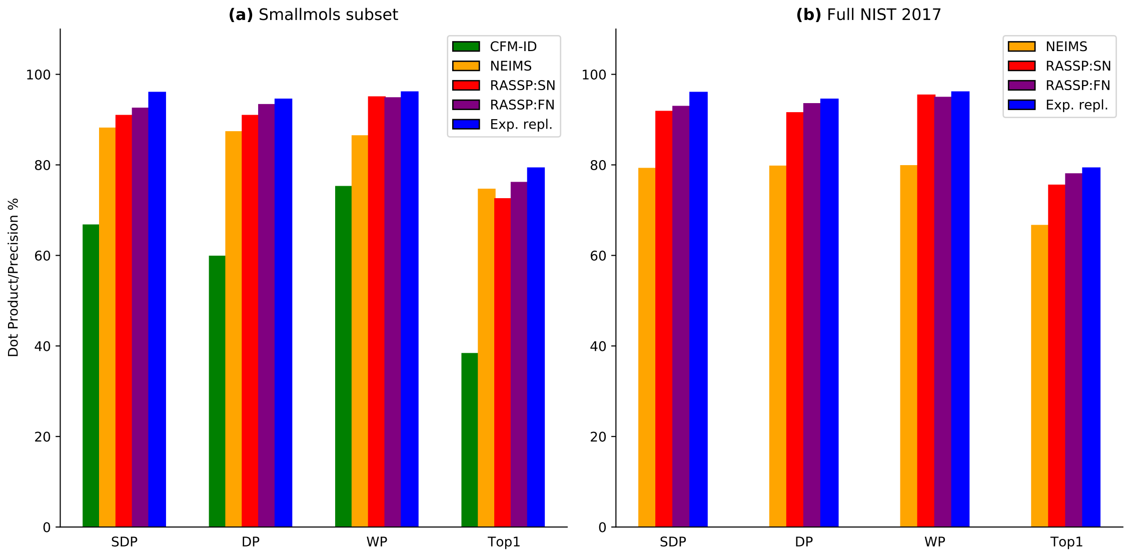

Figure 3: EI-MS prediction performance. Similar to the figure in the Main Text, but the bars here represent the mean value over the entire dataset, and Top-1 accuracy (Top1) is reported instead of Weighted False-Positive Rate (WFPR). Top-1 accuracy was left out of the Main Text due to 10-50-90% percentile reporting failing to display meaningful bars, since Top-1 accuracy is either 0 or 1 for each row (the peak with highest intensity in predicted spectra also matches the peak with highest intensity in the target).

generated via a physically-plausible "break-and-rearrange" process by which all possible bond breakages out to integer depth d are iteratively considered, followed by any possible rearrangement of hydrogens. Running this process on glucose $C_6H_{12}O_6$ outputs 164 unique subsets of the 12 heavy-atoms (6 carbon and 6 oxygen). Considering subsets of all hydrogens is also possible, but makes this process more computationally-intensive.

An example of the atom (vertex) subsets output by our subset enumeration process is shown in Fig. 4. All atom subsets form one connected-component due to our "physically-plausible fragment" assumption. If a bond breakage would generate two separate fragments, then both are considered as separate atom subsets.

Each atom subset maps surjectively onto the set of unique chemical subformulae of the original molecule (each atom subset corresponds to a subformulae, and there can be many subsets that map to the same subformula), and each chemical subformulae gives rise to a unique peak distribution. In Table 5, we see the chemical formula corresponding to each

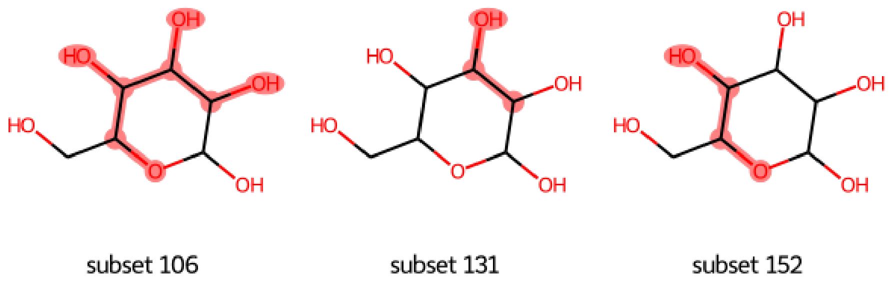

Figure 4: Three randomly-chosen heavy-atom (C and O only) subsets of glucose

subset.

Table 5: Chemical formulae and molecular weight for the three subsets depicted in Fig. 4

Subset idx	Chemical formula	Mol weight
106	C4O4	112.04
131	C2O1	40.02
152	C2O2	56.02

The peak distribution for each of the three subsets in Fig. 4 is shown in Fig. 5. Each peak is shaded according to the intensity. Note the primary peak centered at the exact weight of the molecular ion listed in Table 5, but also the faint echoes of peaks at higher mass, caused by the naturally-occurring isotopic variability of carbon and oxygen.

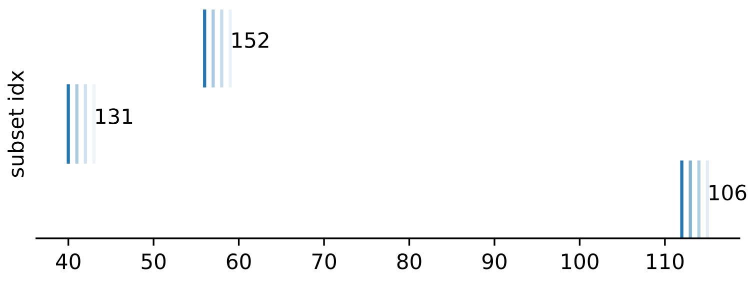

Figure 5: The barcode spectra corresponding to the three subsets depicted in Fig. ??. Each peak is shaded proportionally to the intensity. The X-axis corresponds to Daltons/amu.

Our prediction models are trained to output a probability distribution over subformulae (RASSP:FN) and subsets (RASSP:SN). Once such a probability distribution is obtained, it is a simple matter of scaling the peak distribution corresponding to each subset with the probability for that subset, and then summing all the peak distributions together to get the

final output spectrum.

For sake of completion, we also provide the indicator matrix that describes all 164 heavy-atom subsets in Fig. 6 and the barcode spectrum for each subset in Fig. 7.

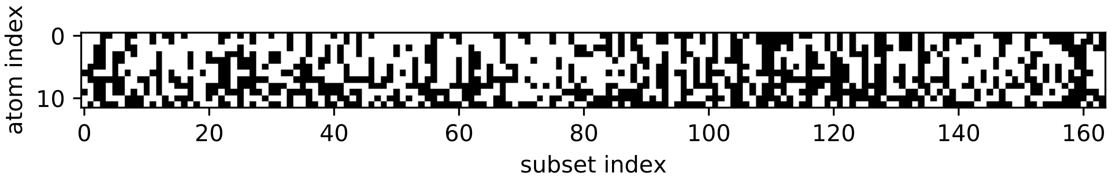

Figure 6: Indicator matrix depicting each of the 164 heavy-atom subsets of glucose produced by our enumeration scheme. The presence of the atom is shown in white, and the absence of the atom is shown in black. The first 6 atom indexes are carbon and the last 6 atom indexes are oxygen.

Toluene example

Toluene is a simple example illustrating the tradeoffs and improvements our spectral prediction process makes.

Fig. 8 illustrates FormulaNet's prediction of the toluene spectrum (negative values, in blue) vs the ground-truth experimental spectrum (positive values, in black). We note that our predicted spectrum captures all the important peaks attributable to fragments in the well-studied fragmentation process at [39, 51, 65, 77, 91, 92] Daltons. We have highlighted these peaks as light vertical lines in red.

We note that the 7-member ring ion featured in the fragmentation process is not a fragment or subgraph explicitly considered in our process, due to computational constraints. Rather, our subset and subformula enumeration process considers both the 91 amu 6-member ring ion with attached carbon (after a single hydrogen loss) and the 7-member ring ion as identical, due to having the same set of underlying atoms. Throwing away bond information in the subset/subformula enumeration process is critical in making our solution computationally feasible, but it does result in losing the ability to separate the 6-member ring and the 7-member ring, even though they present in the mass spectrometer as the same peak.

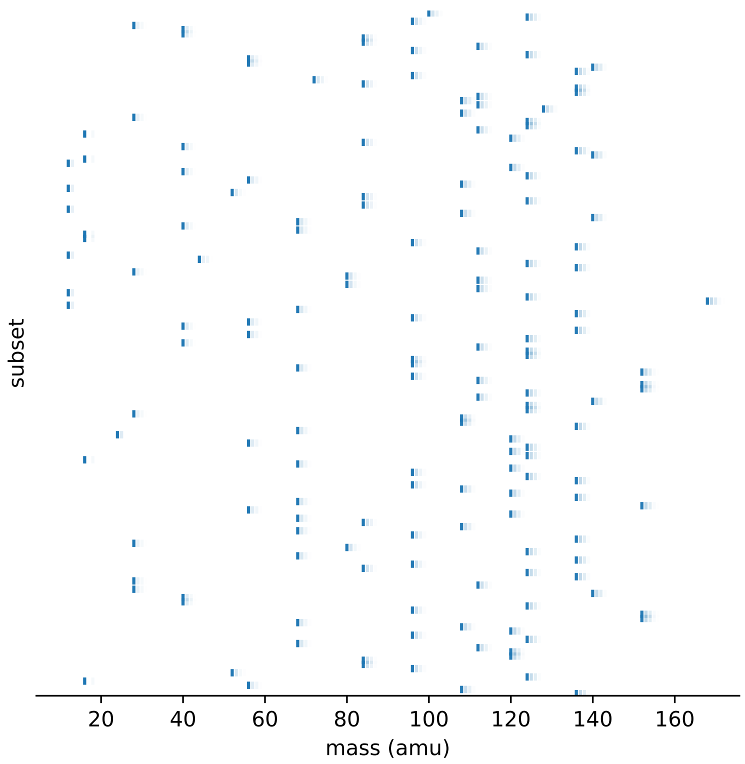

Figure 7: Barcode spectra for each of the 164 heavy-atom subsets of glucose produced by our enumeration scheme.

References

(1) NIST Standard Reference Database 1A. Data version v17, software version 2.XX. `https://www.nist.gov/srd/nist-standard-reference-database-1a`.

(2) Allen, F.; Pon, A.; Wilson, M.; Greiner, R.; Wishart, D. CFM-ID: a web server for annotation, spectrum prediction and metabolite identification from tandem mass spectra. **2014**, 6.

(3) Allen, F.; Pon, A.; Greiner, R.; Wishart, D. Computational Prediction of Electron Ionization Mass Spectra to Assist in GC/MS Compound Identification. *Anal. Chem.* **2016**, 9.

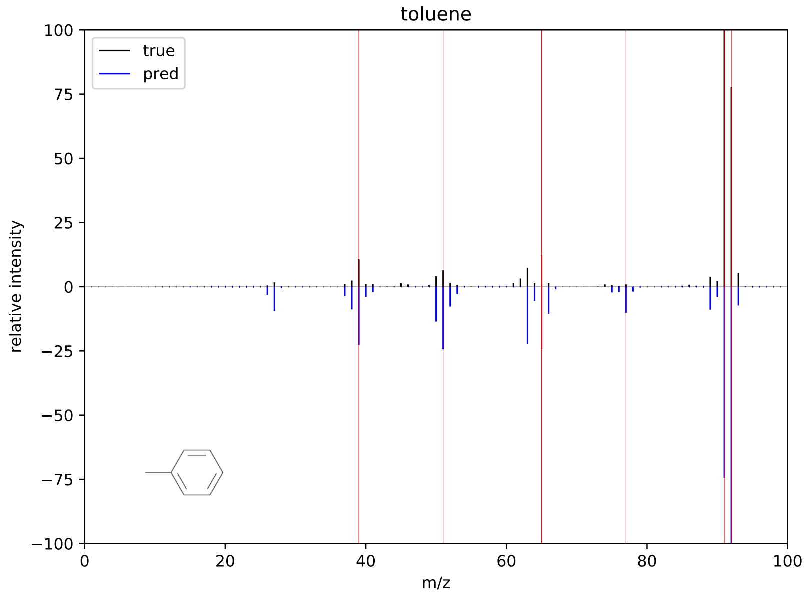

Figure 8: Toluene

(4) Kim, S.; Chen, J.; Cheng, T.; Gindulyte, A.; He, J.; He, S.; Li, Q.; Shoemaker, B. A.; Thiessen, P. A.; Yu, B.; Zaslavsky, L.; Zhang, J.; Bolton, E. E. PubChem in 2021: new data content and improved web interfaces. *Nucleic Acids Research* **2020**, *49*, D1388–D1395, ISBN: 0305-1048 Type: 10.1093/nar/gkaa971.

(5) Sanchez-Lengeling, B.; Reif, E.; Pearce, A.; Wiltschko, A. B. *A Gentle Introduction to Graph Neural Networks*; 2021.

(6) Zhu, H.; Liu, L.; Hassoun, S. *Using Graph Neural Networks for Mass Spectrometry Prediction*; 2020; arXiv:2010.04661 [cs] type: article.

(7) Wei, J. N.; Belanger, D.; Adams, R. P.; Sculley, D. Rapid Prediction of Electron–Ionization Mass Spectrometry Using Neural Networks. *ACS Central Science* **2019**, 9.

(8) Landrum, G. RDKit: Open-Source Cheminformatics Software. `https://www.rdkit.org/`.
